# Polygenic Analysis in Absence of Major Effector *ATF1* Unveils Novel Components in Yeast Flavor Ester Biosynthesis

**DOI:** 10.1128/mBio.01279-18

**Published:** 2018-08-28

**Authors:** Sylvester Holt, Bruna Trindade de Carvalho, María R. Foulquié-Moreno, Johan M. Thevelein

**Affiliations:** a Department of Biology, Laboratory of Molecular Cell Biology, Institute of Botany and Microbiology, KU Leuven, Leuven-Heverlee, Flanders, Belgium; b Center for Microbiology, VIB, Leuven-Heverlee, Flanders, Belgium; Harvard Medical School

**Keywords:** ethanol acetyl-CoA transferase, ethyl acetate, flavor, QTL analysis, alcoholic beverages, brewer's yeast, industrial yeast

## Abstract

Flavor production in yeast fermentation is of paramount importance for industrial production of alcoholic beverages. Although major enzymes of flavor compound biosynthesis have been identified, few specific mutations responsible for strain diversity in flavor production are known. The *ATF1*-encoded alcohol acetyl coenzyme A (acetyl-CoA) transferase (AATase) is responsible for the majority of acetate ester biosynthesis, but other components affecting strain diversity remain unknown. We have performed parallel polygenic analysis of low production of ethyl acetate, a compound with an undesirable solvent-like off-flavor, in strains with and without deletion of *ATF1*. We identified two unique causative mutations, *eat1*^K179*fs*^ and *snf8*^E148^*, not present in any other sequenced yeast strain and responsible for most ethyl acetate produced in absence of *ATF1*. *EAT1* encodes a putative mitochondrial ethanol acetyl-CoA transferase (EATase) and its overexpression, but not that of *EAT1*^K179*fs*^, and strongly increases ethyl acetate without affecting other flavor acetate esters. Unexpectedly, a higher level of acetate esters (including ethyl acetate) was produced when *eat1*^K179*fs*^ was present together with *ATF1* in the same strain, suggesting that the Eat1 and Atf1 enzymes are intertwined. On the other hand, introduction of *snf8*^E148^* lowered ethyl acetate levels also in the presence of *ATF1*, and it affected other aroma compounds, growth, and fermentation as well. Engineering of *snf8*^E148^* in three industrial yeast strains (for production of wine, saké, and ale beer) and fermentation in an application-relevant medium showed a high but strain-dependent potential for flavor enhancement. Our work has identified *EAT1* and *SNF8* as new genetic elements determining ethyl acetate production diversity in yeast strains.

## Abstract (precis)

Basic research with laboratory strains of the yeast Saccharomyces cerevisiae has identified the structural genes of most metabolic enzymes, as well as genes encoding major regulators of metabolism. On the other hand, more recent work on polygenic analysis of yeast biodiversity in natural and industrial yeast strains is revealing novel components of yeast metabolism. A major example is the metabolism of flavor compounds, a particularly important property of industrial yeast strains used for the production of alcoholic beverages. In this work, we have performed polygenic analysis of production of ethyl acetate, an important off-flavor compound in beer and other alcoholic beverages. To increase the chances of identifying novel components, we have used in parallel a wild-type strain and a strain with a deletion of *ATF1* encoding the main enzyme of acetate ester biosynthesis. This revealed a new structural gene, *EAT1*, encoding a putative mitochondrial enzyme, which was recently identified as an ethanol acetyl-CoA transferase in another yeast species. We also identified a novel regulatory gene, *SNF8*, which has not previously been linked to flavor production. Our results show that polygenic analysis of metabolic traits in the absence of major effector genes can reveal novel structural and regulatory genes. The mutant alleles identified can be used to affect the flavor profile in industrial yeast strains for production of alcoholic beverages in more subtle ways than by deletion or overexpression of the already known major effector genes and without significantly altering other industrially important traits. The effect of the novel variants was dependent on the genetic background, with a highly desirable outcome in the flavor profile of an ale brewing yeast.

## INTRODUCTION

Although genetic studies are rapidly contributing to our understanding of the molecular basis of flavor compound production and its regulation, there are still major gaps in our knowledge of enzymes and regulators in specific pathways (1). An important example is ethyl acetate, a compound with a solvent-like off-flavor that negatively affects aroma perception in beer and other alcoholic beverages (2). It has been known for more than 30 years that the alcohol acetyl coenzyme A (acetyl-CoA) transferase (AATase) genes *ATF1* and *ATF2* encode enzymes that are responsible for the majority of acetate ester biosynthesis in the yeast Saccharomyces cerevisiae (3–5). However, an *atf1Δ atf2Δ* deletion strain still produces ethyl acetate at 50% of the wild-type (WT) level, clearly indicating the existence of one or more unknown enzymes (6). Recently, Kruis et al. (7) discovered a novel ethanol acetyl-CoA transferase (EATase) enzyme capable of producing high levels of ethyl acetate from ethanol and acetyl-CoA in Wickerhamomyces anomalus (previously known as Pichia anomala) by performing a BLAST search with the S. cerevisiae genes *ATF1* and *ATF2* against the sequenced W. anomalus genome and expressing candidate genes in S. cerevisiae. Yeast engineered for overexpression of the S. cerevisiae gene *YGR015C*, encoding an ortholog of the W. anomalus EATase, produced more ethyl acetate during aerobic fermentation and the gene was therefore named *EAT1* for ethanol acetyl-CoA transferase 1 (7). This gene is a distant ortholog (<14.5% amino acid identity) of the *EHT1* and *EEB1* genes, previously identified by our research group, that encode ethanol acyl-CoA transferases and produce ethyl esters during alcoholic fermentation with S. cerevisiae (8). *EAT1* has not been investigated in semianaerobic alcoholic fermentations up until now, but is likely to be one of the missing enzymes in ethyl acetate production.

Ethyl acetate production during alcoholic fermentation has been investigated previously by random mutagenesis and pooled-segregant quantitative trait locus (QTL) analysis, with the identification of causative alleles of *TPS1*, *PMA1*, and *CEM1* (9). *CEM1* encodes a mitochondrial β-keto-acyl synthase, a homologue of the fas2 β-keto-acyl synthase subunit, that is required for respiration, but apparently does not affect mitochondrial lipid homeostasis (10). The mechanism of Cem1 action in ethyl acetate biosynthesis therefore remains unclear. Moreover, the production level of other important flavor compounds, like isoamyl acetate with banana flavor and other fruity flavor acetate esters, was not determined. The effects observed on ethyl acetate formation of *TPS1*, *PMA1*, and *CEM1* might therefore have been due to altered activity of Atf1 and/or Atf2.

Pooled-segregant QTL analysis is an unbiased technique that is used in both applied and fundamental yeast studies to identify causative genetic elements (11, 12). It has been used successfully to determine genes underlying several metabolic traits, including production of central metabolites for yeast metabolism (13), glycerol (14), acetic acid (15), sulfite (16), and the aroma compounds 2-phenylethyl acetate (17), isoamyl alcohol, isobutanol, 2-phenyl ethanol, ethyl octanoate and decanoate (18, 19), and 4-sulfanyl-methylpentan-2-one (20). In general, little work has been reported on the introduction of the identified alleles in other strain backgrounds in spite of its importance for evaluating the general applicability of the alleles for industrial strain improvement.

In this work, we first identified a strain with low ethyl acetate and average isoamyl acetate production, since this provides a good marker for *in vivo* Atf1 and Atf2 enzyme activity (4, 21–23). We then performed parallel polygenic analysis in the absence and presence of the major AATase *ATF1* gene to identify novel genetic elements responsible for the remaining ethyl acetate production. This enabled us to identify variants in the genes *EAT1* and *SNF8*, as affecting flavor production by S. cerevisiae. With the identification of *EAT1* as a causative genetic element, we have demonstrated that QTL mapping can be used successfully to identify new structural genes encoding metabolic pathway enzymes. The variants identified in *EAT1* and *SNF8* were responsible for 75% of the variation between the parental strains in the absence of the *ATF1* gene. However, when engineered in the *ATF1* wild-type background, the *ATF1*-derived acetate ester productivity was altered in different ways by the two mutant alleles. These effects occurred in a strain-dependent fashion, underscoring the necessity of introducing natural variants in unrelated genetic backgrounds to validate their applicability in industrial yeasts.

## RESULTS

### Screening of a diverse yeast strain collection.

**FIG 1:**
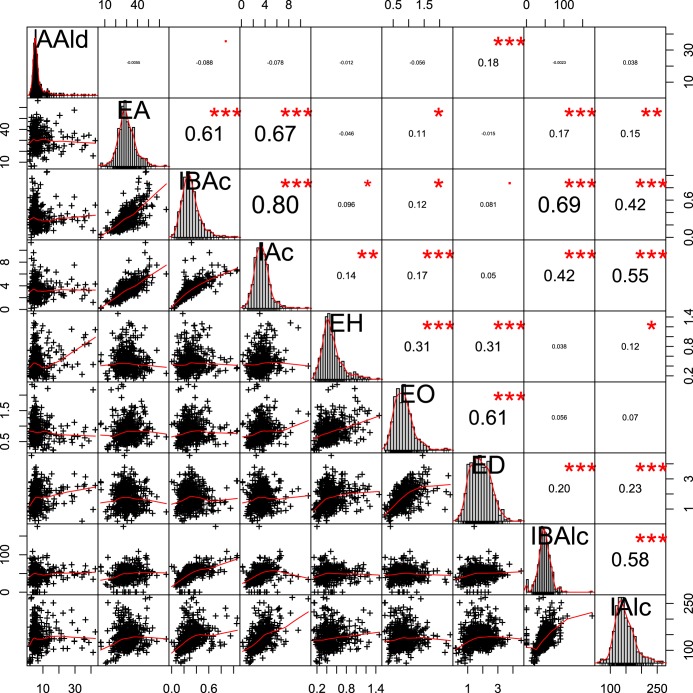
Distribution and correlations between the aroma production values in strains from the MCB collection. The aroma profile of 429 *Saccharomyces* sp. strains isolated from diverse sources (part of the MCB collection) was analyzed using GC-FID in fermentations with YP250–10% glucose medium. Abbreviations for compounds: AAld, acetaldehyde; EA, ethyl acetate; IBAc, isobutylacetate; EH, ethyl hexanoate; EO, ethyl octanoate; ED, ethyl decanoate; IBAlc, isobutyl alcohol; IAlc, isoamyl alcohol. Correlation coefficients between the aroma compound levels and distributions of the production range are shown. The figure contains scatter plots, histograms with smoothed fits, and density plots (red lines). The correlations were calculated with Spearman correlation coefficients, and *P* values are indicated as follows: *, *P* ≤ 0.05; **, *P* ≤ 0.01; ***, *P* ≤ 0.001. The analysis was performed with the R package PerformanceAnalytics.

**FIG 2:**
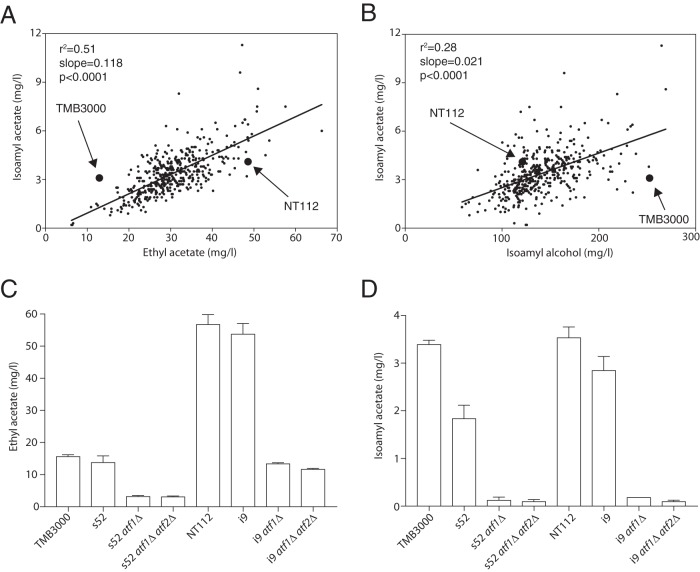
Screening for superior and inferior isoamyl acetate/ethyl acetate ratios in parent strains. (A and B) Scatter plot of ethyl acetate versus isoamyl acetate production levels (A) and isoamyl alcohol and isoamyl acetate production levels (B). The positions of the low-ethyl-acetate-producing strain TMB 3000 and high-ethyl-acetate-producing wine strain Anchor NT112 are indicated on the plot. The normality of ethyl acetate, isoamyl acetate, and isoamyl alcohol production was confirmed with QQ plots and the linear regression line is shown in the figure. (C and D) Ethyl acetate (C) and isoamyl acetate (D) production levels in the superior strain TMB 3000 and its s52 segregant and in the inferior strain NT112 and its i9 segregant and in the same strains containing *atf1Δ* or *atf1Δ atf2Δ*. Fermentations were carried out in synthetic yeast extract-Bacto peptone medium with a free amino nitrogen content of 250 mg/liter at pH 4.5 and a temperature of 25°C.

We have performed small tube fermentations with YPD (yeast extract-peptone-dextrose) as the substrate using a yeast collection consisting of 429 *Saccharomyces* sp. strains used for production of foodstuffs or alcoholic beverages, such as wine and beer, or isolated from natural sources (from the MCB yeast collection [see Materials and Methods for details]), and we quantified the major volatile aroma compounds. The volatile metabolites showed considerable variation for all measured aroma compounds, indicating complex genetic variation effects on their metabolic pathways (Fig. 1). Ethyl acetate levels were closely correlated with those of isoamyl acetate (Fig. 1 and 2A) (Pearson’s *r*^2^ = 0.51), as well as with those of isobutyl acetate (Fig. 1), as previously described (24). From the screening results, we have identified a Saccharomyces cerevisiae strain, TMB 3000 (ATCC 96581), originating from a spent sulfite liquor fermentation plant in Sweden (25), with a very low ethyl acetate production of 12.9 mg/liter (versus the strain collection average ± standard deviation [SD] of 30.1 ± 3.8 mg/liter; *n =* 429) but a regular isoamyl acetate production of 3.1 mg/liter (versus the strain collection average of 3.3 ± 0.7 mg/liter; *n =* 429) (Fig. 2A). The TMB 3000 strain also produced 252.4 mg/liter isoamyl alcohol, which is considerably higher than the average of the yeast strains (124.8 ± 15.3 mg/liter; *n =* 429) (Fig. 2B), suggesting that the low ethyl acetate production was at least to some extent due to lower AATase enzyme activity.

### Generation of haploid parent strains.

**FIG 3:**
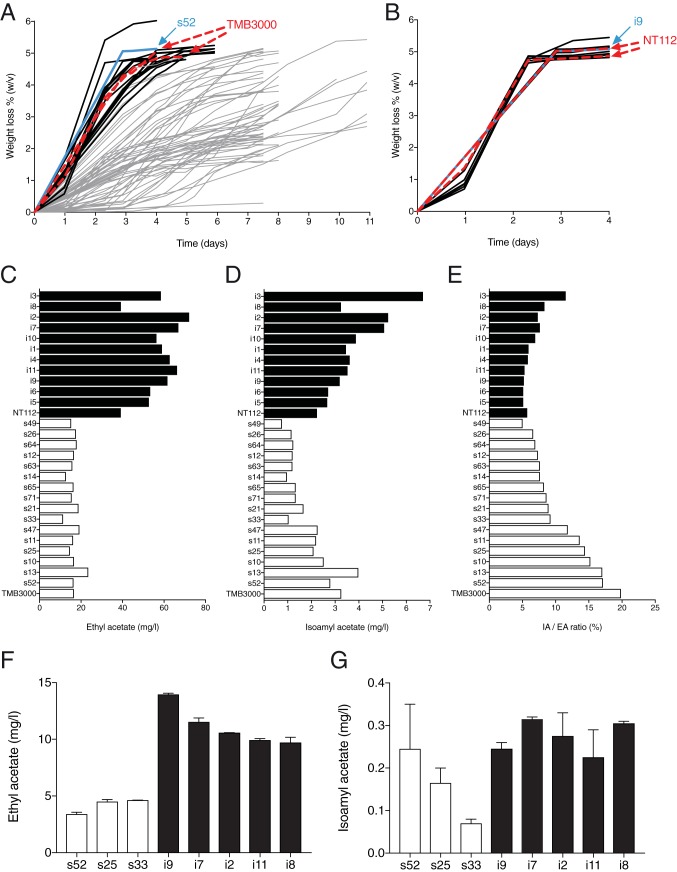
Identification of segregants showing complete fermentation and low production of ethyl acetate for polygenic analysis with TMB 3000 and NT112. Offspring from (A) TMB 3000 (76 segregants) and (B) NT112 (11 segregants) were assayed for fermentation capacity. Fermentations were done in two different batches, in which the parent TMB 3000 and NT112 strains were each time included (indicated by bold dashed red lines). The superior (s52) and inferior (i9) segregants used for QTL analysis are indicated by bold blue lines. Black lines indicate strains leaving less than 2 g/liter residual glucose at the end of the fermentation, considered to be a complete fermentation. The production of (C) ethyl acetate and (D) isoamyl acetate, as well as (E) the isoamyl acetate/ethyl acetate ratio (in percentage), was subsequently analyzed in the segregants showing a fermentation profile similar to that of the TMB 3000 and NT112 strains. The *ATF1* gene was deleted in segregants with high or low production of ethyl acetate and a high or low isoamyl acetate/ethyl acetate ratio. The production of (F) ethyl acetate and (G) isoamyl acetate is shown for the *atf1Δ* strains of the selected segregants.

Strain TMB 3000 (ATCC 96581) and the Anchor S. cerevisiae wine yeast strain NT112, which showed high production of ethyl acetate, were both sporulated, and their offspring were first assayed for fermentation capacity (Fig. 3A and B). In segregants exhibiting a fermentation profile similar to that of the parental strains, we determined the production of ethyl acetate (Fig. 3C), isoamyl acetate (Fig. 3D) and the isoamyl acetate/ethyl acetate (IA/EA) ratio (Fig. 3E). Next, the *ATF1* gene was deleted in segregants with the lowest (three strains) or the highest (five strains) ethyl acetate production levels and a high or low IA/EA ratio. The production of ethyl acetate (Fig. 3F) and isoamyl acetate (Fig. 3G) is shown for the *atf1Δ* strains of these selected segregants.

The superior segregant 52 (s52) and the inferior segregant 9 (i9) showed, in the absence of the *ATF1* gene, production levels of ethyl acetate of 3.3 and 13.5 mg/liter, respectively, and since additional deletion of the *ATF2* gene had little effect (Fig. 2C), novel genetic elements had to be responsible for the conspicuous difference between the two strains. Deletion of the *ATF2* gene caused a minor decrease in isoamyl acetate, significant for the i9 segregant (0.09 ± 0.01 mg/liter; *n =* 3; *P* = 0.003 [Fig. 2D]), which is negligible compared to its aroma threshold of 1.6 mg/liter in beer (26). This decrease observed in the i9 strain is much smaller than that previously reported for fermentation of an auxotrophic laboratory strain using standard YPD medium (6). Due to the very minor effects of *ATF2* deletion, we have disregarded the Atf2 enzyme from further analysis.

**FIG 4:**
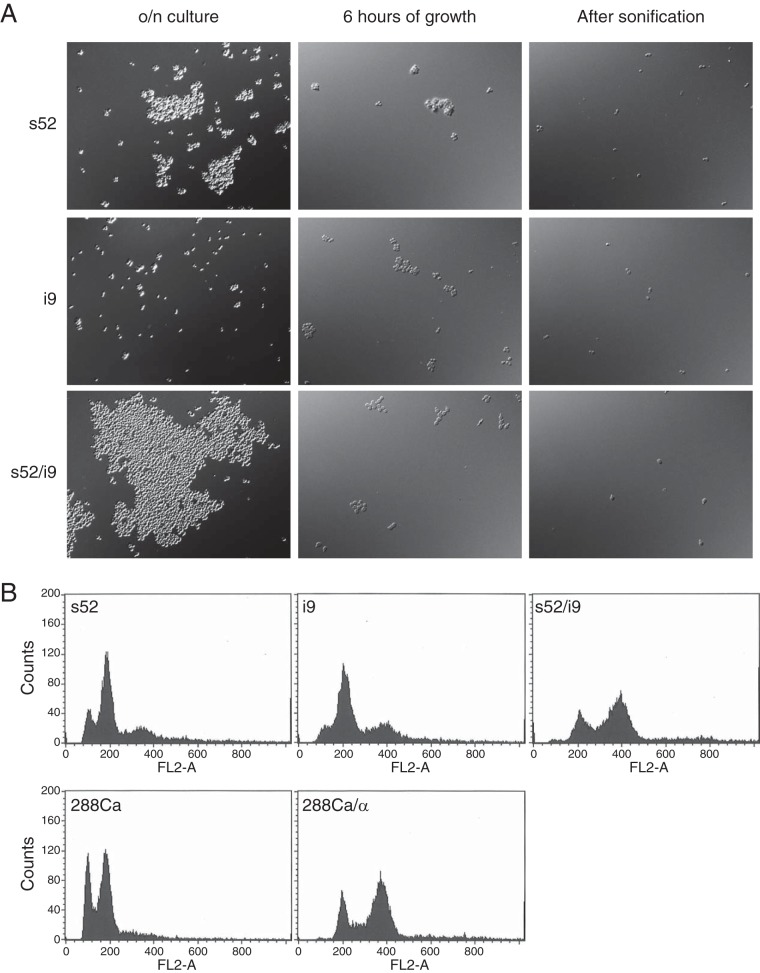
Dispersal of cell flocs and confirmation of ploidy. Dispersal of cell clumps was performed by sonication in exponentially growing cultures. (A) Treatment of cell aggregates of the s52 and i9 strains to obtain single cells. o/n, overnight. (B) Flow cytometry of sonicated cells after DNA staining with propidium iodide. The first two peaks indicate the number of cells with a DNA content corresponding to that before (1n; G_1_ phase) and after (2n; G_2_ phase) duplication of the genome.

The low-ethyl-acetate-producing TMB 3000 yeast strain and its segregants exhibited heavy cell aggregation and formed large flocs in stationary-phase cultures. For the s52 segregant and also for the high-ethyl-acetate-producing i9 segregant, sonication treatment led to a mixture mainly consisting of single cells and 2-cell aggregates, whereas it caused complete dispersal of the hybrid s52/i9 strain into single cells (Fig. 4A). This allowed us to confirm the haploid character of the segregants and the diploid character of the s52/i9 hybrid (Fig. 4B).

### QTL mapping by pooled-segregant whole-genome sequence analysis.

**FIG 5:**
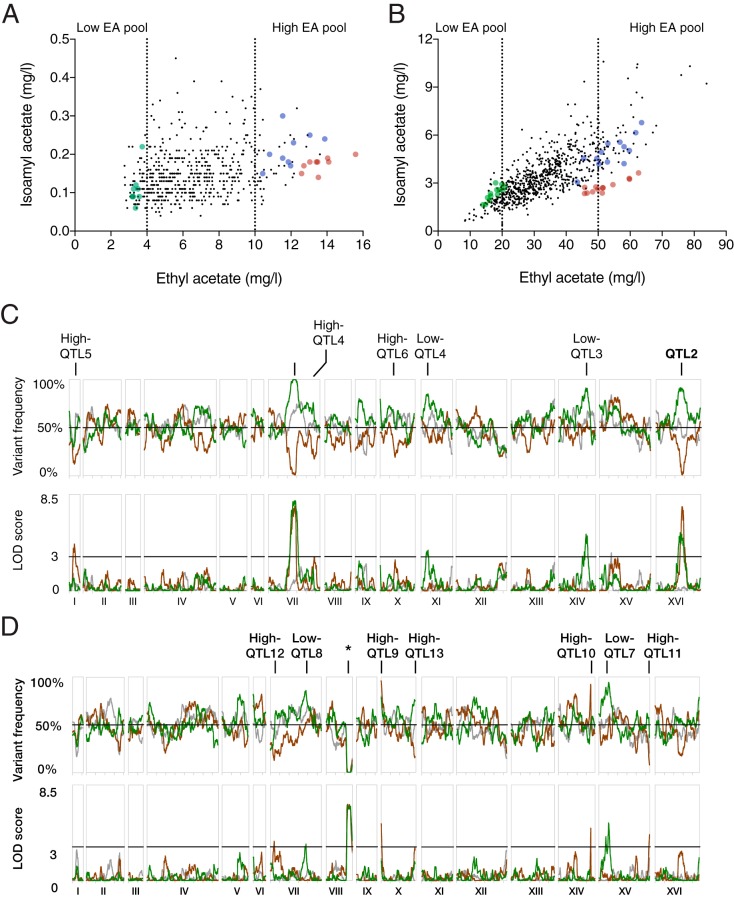
Pooled-segregant QTL analysis in the presence and absence of the major AATase gene *ATF1*. The aroma profile of (A) 507 *atf1*Δ segregants and (B) 713 WT *ATF1* haploid segregants was determined in small tube fermentations using GC-FID. The parent strains s52 (green), i9 (red), and diploid hybrids (blue) were included in all fermentation batches. Pooled-segregant QTL analysis was performed with (C) *atf1*Δ and (D) WT *ATF1* segregants using pools of low (green)-, high (red)-, and random (gray)-ethyl acetate-producing segregants. The SNP allele variant frequency refers to the percentage of the SNP nucleotide in the pool originating from the s52 low-ethyl-acetate-producing strain. A cutoff of 3 for the LOD score (1,000:1 odds ratio in favor of a nonrandom event) was used to determine significant deviation in the linkage disequilibrium as indicated. An asterisk indicates a genomic region with full inheritance from the inferior i9 parent in the low-, high-, and randomly selected ethyl acetate-production WT *ATF1* pools.

Hybrids with and without *ATF1* (s52/i9 WT and s52/i9 *atf1*Δ/*atf1*Δ) were sporulated, and their segregants were subjected to fermentation with aroma metabolite quantification by gas chromatography-flame ionization detection (GC-FID). Segregants from both crosses showed a gradual segregation of ethyl acetate production. A strong correlation was observed between ethyl acetate and isoamyl acetate levels in the WT *ATF1* offspring, whereas the production of these two compounds was uncoupled in the *atf1*Δ offspring (Fig. 5A and B). The high-ethyl-acetate-production trait was dominant, as the hybrid strains, s52/i9 WT and s52/i9 *atf1*Δ/*atf1*Δ, produced a similar level of ethyl acetate to the i9 parental haploids, i9 WT and i9 *atf1Δ*. Three pools of segregants were assembled containing strains with low, high, or random production of ethyl acetate. The genomic DNA of the pools was isolated and sequenced, and small sequence variants (single nucleotide polymorphisms [SNPs]) were used to calculate linkage probabilities (logarithm of odds [LOD] scores) along the genome (Fig. 5C and D). At position 430583 of chromosome 8 (inside the gene *PRP8*) of the WT *ATF1* pools, we observed an abrupt loss of heterozygosity with full inheritance of the i9 parent DNA until the end of the chromosome (Fig. 5D). The sequencing coverage remained the same, suggesting loss of s52 DNA and a duplication of i9 DNA during hybrid formation.

Two strongly linked QTL, QTL1, and QTL2, were observed in the *atf1*Δ segregant pools (Fig. 5C). QTL1 and QTL2 were the only QTL that appeared as mirror images in both pools for high or low production of ethyl acetate (Fig. 5C and D; Table 1). Strikingly, neither of the two QTL overlapped the QTL identified in the WT *ATF1* pools. This suggests a high degree of epistasis in yeast ethyl acetate production by the *ATF1* gene product.

**TABLE 1:** QTL involved in ethyl acetate production in the presence and absence of the *ATF1* gene^a^

QTL	EA pool	Segregant linked to	Chromosome, position (kb)	Size (kb)	Maximum LOD score	Maximum/minimum frequency (%)
*atf1*Δ						
QTL1	Low	s52	chrVII, 509.7–591.0	81.3	8.0	99
QTL2	Low	s52	chrXVI, 484.2–570.0	85.8	5.2	92
Low-QTL3	Low	s52	chrXIV, 565.2–613.6	48.4	5.0	92
Low-QTL4	Low	s52	chrXI, 102.5–163.9	61.4	3.6	86
QTL1	High	i9	chrVII, 504.6–580.4	75.8	7.5	1
QTL2	High	i9	chrXVI, 536.6–569.4	32.8	7.5	1
High-QTL5	High	i9	chrI, 82.1–129.3	47.2	4.1	12
High-QTL6	High	i9	chrVII, 952.6–999.0	46.4	3.0	18
High-QTL7	High	i9	chrX, 301.8–345.3	43.5	2.8	18
WT						
Low-QTL8	Low	s52	chrXV, 204.6–224.8	20.2	5.2	94
Low-QTL9	Low	s52	chrVII, 733.8–772.3	38.5	3.3	88
High-QTL10	High	s52	chrX, 0–9.8	9.8	5.0	96
High-QTL11	High	s52	chrXIV, 689.9–694.5	4.6	4.7	93
High-QTL12	High	i9	chrXV, 1,072.2–1,091.291	19.1	4.1	8
High-QTL13	High	i9	chrVII, 65.0–95.5	30.5	3.5	12
High-QTL14	High	i9	chrX, 703.5–745.751	42.3	3.4	15

a The positions of the QTL indicate the 1-LOD drop interval predicted with MULTIPOOL.

### Identification of causative alleles and causative SNPs.

**FIG 6:**
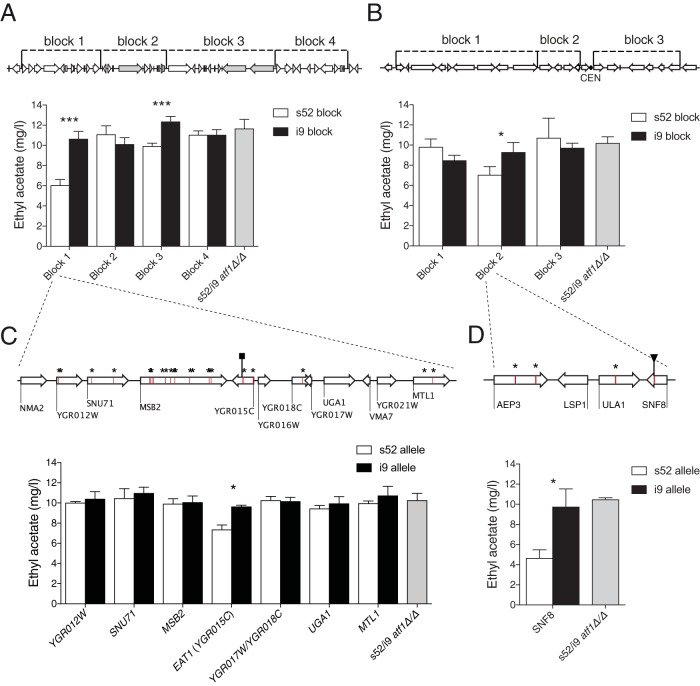
Identification of *EAT1* and *SNF8* as causative genes in QTL1 and QTL2, respectively. RHA of the major QTL QTL1 and QTL2 in the *atf1*Δ pools. These QTL were strong and broad, with the most strongly linked regions being 81 and 76 kbp in QTL1 and 86 and 33 kbp in QTL2, in the low-and high-ethyl-acetate pools, respectively. In QTL2, we used the linkage overlap between the two pools to focus on the most strongly linked area around the centromere in chromosome 16. To further narrow down the causative region(s), we have performed bulk RHA in blocks of about 20 kb. Bulk RHA results are shown in panel A for QTL1 and in panel B for QTL2. The position of the blocks is indicated on top of the figure. To identify the causative genetic elements, individual gene RHA was performed in panel C for QTL1 block 1 and in panel D for QTL2 block 2. Genes with missense, nonsense, or frameshift mutations were prioritized. These mutations are indicated with stars for missense, squares for frameshift, and triangles for nonsense mutations. All mutations in regions with linkage are listed in Table S2.

Using reciprocal hemizygosity analysis (RHA), we have identified the major causative gene in QTL1 as *YGR015C* (Fig. 6A and C). It encodes a mitochondrial protein with previously unknown function but recently identified as an ethanol acetyltransferase enzyme and hence designated *EAT1* (7). The mutant *EAT1* allele, causative for low ethyl acetate production, contains a single nucleotide deletion, 532delA (K179*fs*), causing a frameshift with an early truncation of the gene product as a result. Hence, the mutant *EAT1* allele presumably encodes an inactive gene product. QTL1 also contained a minor, but significant, causative region in block 3 (Fig. 6A). However, we could not identify any single, clearly causative genetic element, in the low- nor in the high-ethyl-acetate-producing parent, by deletion of single genes from this region in the RHA hybrid parent strain (see Fig. S1 in the supplemental material). In the second strong QTL, QTL2, we have used the linkage overlap in the high- and low-ethyl-acetate-production pools to narrow down the most strongly linked area for further detailed investigation. We deleted gene blocks flanking the chromosome 16 centromere located in QTL2. The RHA analysis revealed the causative gene in QTL2 to be *SNF8* (Fig. 6B and D), a gene previously shown to be involved in *SUC2* derepression (27) and of which the gene product also functions as part of the endosomal complex required for targeting (ESCRT II) (28). This *SNF8* allele, causative of low ethyl acetate production in the s52 parent strain, contained a nonsense mutation, G442T, causing an early termination of the gene product (E148*). A BLAST search revealed that both mutations *eat1*^K179*fs*^ and *snf8*^E148^* were unique and were not found in any previously sequenced yeast genome.

10.1128/mBio.01279-18.1 FIG S1  RHA analysis for ethyl acetate production of all genes present in block 3 of QTL1. Download FIG S1, PDF file, 0.2 MB. Copyright © 2018 Holt et al. 2018 Holt et al. This content is distributed under the terms of the Creative Commons Attribution 4.0 International license.

### Engineering of *EAT1* and *SNF8* in i9 and s52 strains.

**FIG 7:**
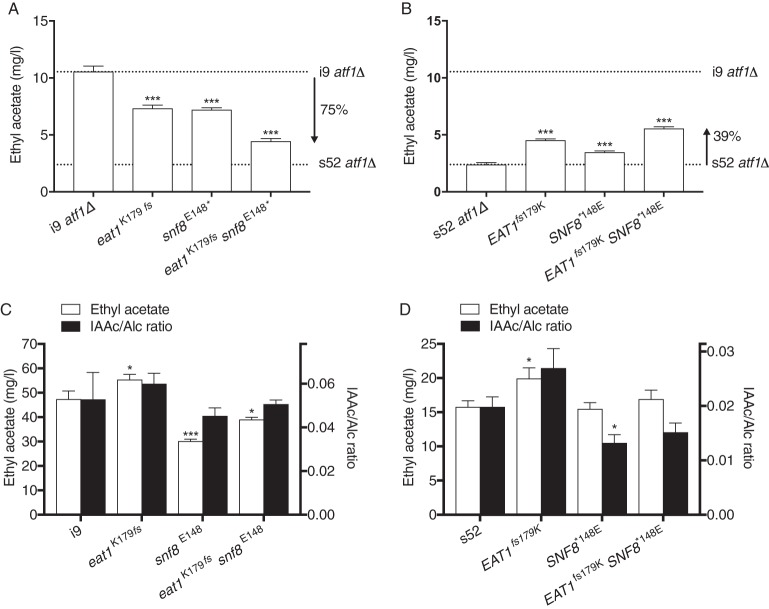
Confirmation of the causative SNPs in *EAT1* and *SNF8*. The causative effects on ethyl acetate production of the *EAT1* frameshift mutation, *eat1*^K179*fs*^ (532delA), and the nonsense *SNF8* mutation, *snf8*^148^* (G442T), were confirmed by exchange of the SNPs between the i9 and s52 parent strains. The ethyl acetate production profile of the engineered strains is shown in panels A for i9 *atf1*Δ, B for s52 *atf1*Δ, C for i9, and D for s52. The isoamyl acetate/isoamyl alcohol ratios are also indicated (black bars, right *y* axis) for the strains containing a WT *ATF1* allele (i9 in panel C and s52 in panel D). All strains were cultured freshly on solid medium with YP–4% glycerol to ensure maintenance of active mitochondrial function, and fermentations were inoculated from cultures grown overnight in YP250–2% glucose. The complete aroma profile is available in Table S3. Significance indications refer to the differences in production levels between the engineered strains and the respective parent strains.

Next, we introduced the *EAT1* frameshift (532delA) and the *SNF8* nonsense (G442T) mutations into the high-ethyl-acetate-producing segregant i9 and the corresponding wild-type sequences into the low-ethyl-acetate-producing segregant s52, in either the WT *ATF1* or *atf1Δ* background, using CRISPR/Cas9 technology (i.e., clustered regularly interspaced short palindromic repeats with Cas9). The i9 *atf1Δ eat1*^K179*fs*^ *snf8*^E148^* and s52 *atf1Δ EAT1 SNF8* strains showed the expected drop or increase in ethyl acetate production, respectively. This resulted in 75% and 39% recovery of the parental phenotypes for i9 and s52, respectively, showing that we had identified the two single nucleotide mutations responsible for the major part of the superior trait of low ethyl acetate production, whereas additional mutations are required for the inferior trait of high ethyl acetate production (Fig. 7).

Higher alcohols were also affected by introduction or restoration of the disruptive mutations in the *EAT1* and *SNF8* genes. Exchange of *snf8*^E148^* into i9 *atf1*Δ also caused a significant increase in acetaldehyde levels, whereas introduction of WT *SNF8* in s52 *atf1*Δ caused a 2-fold increase in higher alcohols (isoamyl alcohol above 300 mg/liter [see Table S2B in the supplemental material]).

10.1128/mBio.01279-18.3 TABLE S1  Annotated mutations in the QTL. All mutations in the QTL identified are shown with reference to the open reading frames. Linkage intervals (1-LOD drop intervals) for low- or high-ethyl-acetate pools and the position of the blocks investigated with RHA analysis are indicated with gray boxes. The mutations refer to differences between the i9 and s52 strains (i9→s52). Genes with structural mutations (nonsense, frameshift, structural variants, etc.), missense mutations, and mutations in the promoter (Pro.) or terminator (Ter.) regions of genes are indicated. SNVs and structural variants inside the QTL were checked manually in the assemblies with IGV. Predicted deletions in *MSB2* (in both s52 and i9) and *TPO2* (in i9) based on a subset of reads with abnormal insert sizes were disregarded. Biallelic SNVs, as well as a small number of multiallelic SNVs, in the haploid assemblies of the s52 and i9 strains were disregarded in the following QTL: QTL1, block 1, *MSB2*; QTL2, *YNL019*, *RQC2*, *YNL017C*, *YKL163W*, *YGR130C*, *YGR138C*, and *YGR122C-A*; High-QTL8, *FDH1*, *FEX1*, *YOR390W*, *YOR392W*, *PAU21*, and *YOR396C-A*; High-QTL9, *SAP4* (K528 [1575delT]). *RPL17A* contained 3 mutations in the intron located within the gene. Download TABLE S1, DOCX file, 0.1 MB. Copyright © 2018 Holt et al. 2018 Holt et al. This content is distributed under the terms of the Creative Commons Attribution 4.0 International license.

10.1128/mBio.01279-18.4 TABLE S2  Aroma compounds produced in fermentations by strains used to confirm the causative frameshift and nonsense mutations in *EAT1* and *SNF8*. Shown are aroma profiles produced in fermentations by the (A) i9 *atf1*Δ, (B) s52 *atf1*Δ, (C) i9, and (D) s52 strains engineered with *EAT1* frameshift and *SNF8* nonsense mutations. The significance of any deviation from the mean of the parent or the engineered strains is indicated as follows: ns, not significant (*P* > 0.05); *, *P* ≤ 0.05; **, *P* ≤ 0.01; ***, *P* ≤ 0.001; Bd, below detection. All fermentations were carried out with four replicates, and aroma production values are shown ± SD. Download TABLE S2, DOCX file, 0.1 MB. Copyright © 2018 Holt et al. 2018 Holt et al. This content is distributed under the terms of the Creative Commons Attribution 4.0 International license.

### Engineering of *EAT1* and *SNF8* in WT *ATF1* strains.

Apart from the elevated levels of acetaldehyde, which was consistently observed in all engineered i9 strains, multiple differences were observed between strains with and without *ATF1* (Table S2). Surprisingly, we observed an increase in ethyl acetate in the presence of *ATF1* in the haploid i9 strain upon introduction of the *eat1*^E179*fs*^ mutation (Fig. 7C). This increase was associated with a significant increase in isoamyl acetate, which was higher than the increase in isoamyl alcohol. Consequently, the isoamyl acetate/alcohol (IAAc/Alc) ratio was increased by 12%, although this difference was not statistically significant (Fig. 7C; Table S2C). On the other hand, the introduction of *snf8*^K148^* in i9 significantly lowered ethyl acetate production (Fig. 7C). This indicates the commercial potential of engineering the identified *snf8*^K148^* mutation into WT *ATF1* strains, in order to obtain a superior ratio of isoamyl acetate to ethyl acetate (fruitier, less solvent-like).

### *snf8*^E148^* affects fermentation capacity and growth with di- and trisaccharides.

**FIG 8:**
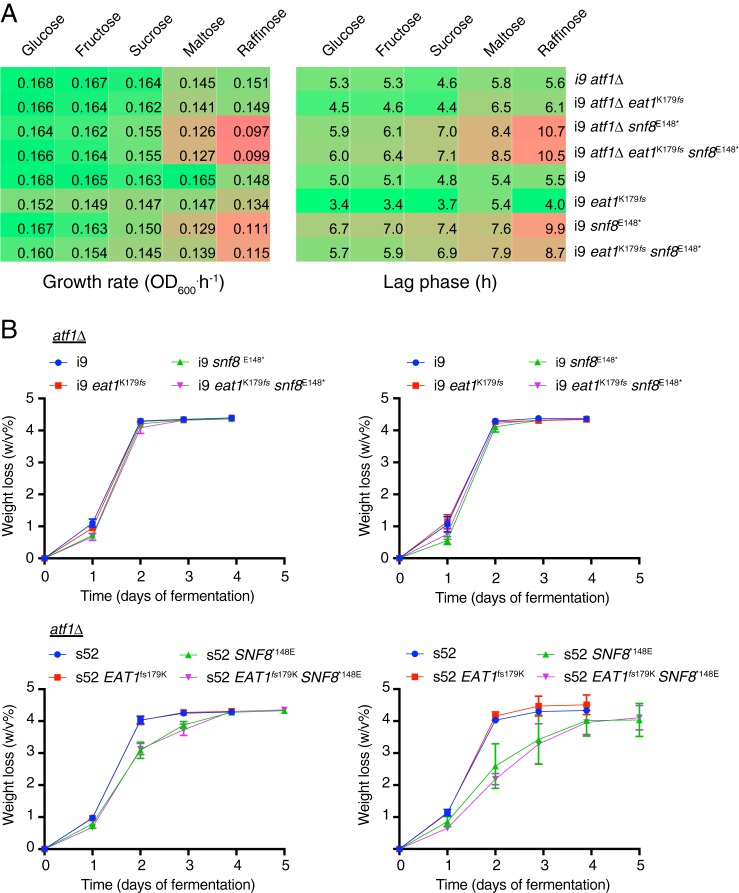
Growth and fermentation rate of the i9 and s52 strains engineered with *EAT1* frameshift and *SNF8* nonsense mutations. Growth kinetics was only analyzed for the i9 strains due to the heavy cell aggregation of the s52 strains. (A) Maximal growth rate and lag phase of the engineered i9 strains were determined from growth curves with the R package Grofit. For growth experiments, the cells were precultured overnight in YP250–2% glucose, washed with YP250 without sugar, and inoculated into YP250 with 2% of the indicated carbon source at a starting OD_600_ of 0.1. (B) Fermentation profiles of i9 *atf1*Δ, s52 *atf1*Δ, i9, and s52 in YP250–10% glucose. Weight loss of the fermentation tubes due to CO_2_ release was used to determine fermentation progress. The fermentations were carried out as described in Materials and Methods.

Mutant *SNF8* alleles were first identified in a screen for deficient growth on raffinose, which was confirmed later upon disruption of the entire open reading frame (27, 29). The *SNF* mutants generally have impaired growth on sucrose, hence the name “sucrose nonfermenting 8” (*SNF8*). However, the mutations in *SNF8* only caused a partial repression of the *SUC2* invertase gene, and the name was kept for historical reasons (27). The introduction of the *snf8*^E148^* mutation in the i9 strains caused a longer lag phase for growth with glucose, fructose, sucrose, and raffinose, which was most pronounced with raffinose (Fig. 8A). The completion of fermentation with glucose was also delayed in the i9 strains containing the *snf8*^E148^* mutation and in the s52 strains containing SNF8*^148E^ compared to the controls without the mutation (Fig. 8B).

### A new enzyme for ethyl acetate biosynthesis.

**FIG 9:**
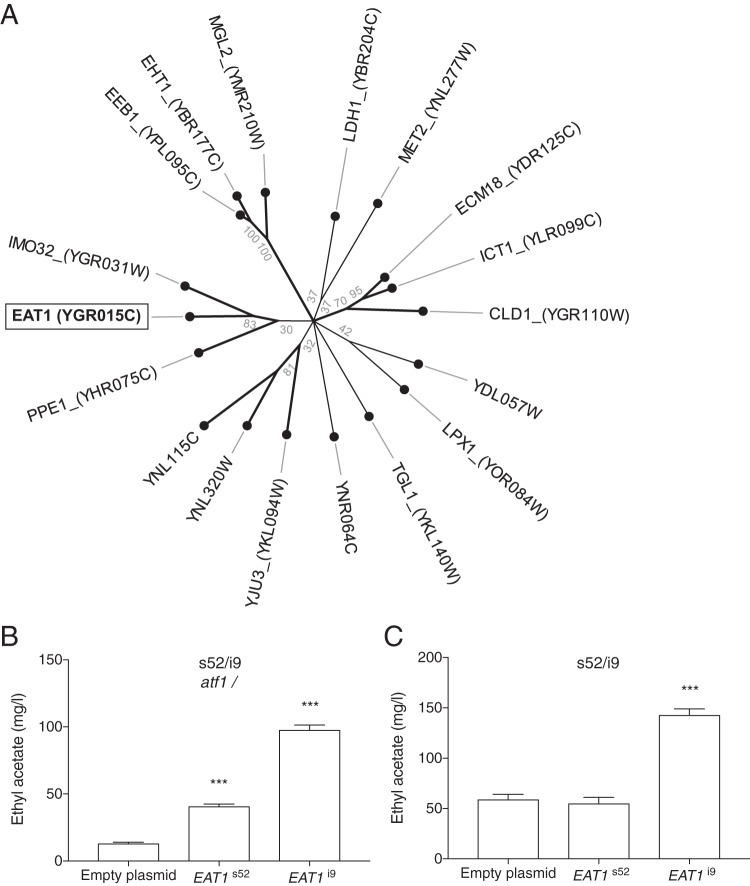
Phylogeny of S. cerevisiae *EAT1* paralogs and effect on ethyl acetate production of *EAT1* overexpression. (A) Phylogenetic tree of S. cerevisiae Eat1 paralogs. A sequence similarity search performed with HMMER (53) identified significant sequence similarity to 18 S. cerevisiae proteins containing α/β-hydrolase 1 (accession no. PF00561.18, amino acid residues 39 to 309; E value, 2.9 × 10^−33^), α/β-hydrolase 6 (accession no. PF12697.5; amino acid residues 42 to 313; E value, 7.2 × 10^−10^), and hydrolase 4 (accession no. PF12146.6; amino acid residues 36 to 194; E value, 1.8 × 10^−06^) domains. The domain sequence of these proteins was aligned with Clustal Omega, and a neighbor-joining phylogenetic tree was made with CLC workbench. Bootstrap analysis was performed with 500 replicates and used to collapse branches with confidence below 30% and to highlight branching points with confidence higher than 70%. The *EAT1* allele originating from the s52 low- or i9 high-ethyl-acetate-producing strains was overexpressed in the s52/i9 hybrids either without *ATF1* (s52/i9 *atf1*Δ/Δ) (B) or with *ATF1* (s52/i9) (C).

The *EAT1* gene encodes a putative ethanol acetyl-CoA transferase, but its relationship to other classes of enzymes has remained unclear. We have investigated by sequence comparison phylogenetic relationships between Eat1 and other enzymes encoded in the genome of S. cerevisiae. The *EAT1* gene product gave 17 significant hits to proteins with α/β-hydrolase 1 and 6 and hydrolase 4 domains (Fig. 9A). These included the previously known ester biosynthesis enzymes Eht1, Eeb1, and Ymr210w (8) with 12.5 to 14.5% identical amino acid residues inside the α/β-hydrolase domain. The closest relative to Eat1 is Imo32 (with 23% identical amino acid residues inside the α/β-hydrolase domain). The *IMO32* gene encodes another putative mitochondrial enzyme of unknown function. To further assess a possible role for both gene products in flavor production, we overexpressed the s52- and i9-derived alleles of both *EAT1* and *IMO32* using the multicopy plasmid p426 and the constitutive TEF1 promoter in the s52/i9 *atf1*Δ/Δ and s52/i9 hybrids. Overexpression of the superior s52 *EAT1* allele led to an increase with 28 mg/liter of the ethyl acetate level, but only in the absence of *ATF1*, suggesting overlapping functions for the two enzymes (Fig. 9B and C). On the other hand, overexpression of the inferior i9 allele of *EAT1* yielded a much higher increase in ethyl acetate production with about 85 mg/liter in both the presence and in the absence of *ATF1* (Fig. 9B and C). In the presence of *ATF1*, this increase was specific for ethyl acetate (see Table S3B in the supplemental material), indicating that the high levels of ethyl acetate with its undesirable solvent-like off-flavor produced by specific yeast strains might be due at least in part to higher activity of the *EAT1* gene product. Therefore, downregulation of Eat1 activity might constitute a possible approach to diminish solvent-like off-flavor caused by high ethyl acetate levels.

10.1128/mBio.01279-18.5 TABLE S3  Aroma compounds produced by strains with overexpression of *EAT1* and *IMO32* from the multicopy p426 plasmid and the constitutive *TEF1* promoter. The *EAT1* and *IMO32* genes were PCR amplified from the s52 or i9 strains and cloned into the multicopy p426 plasmid under a constitutive TEF1 promoter. The production of aroma metabolites was analyzed by GC-FID in the hybrid strains (A) without *ATF1* (s52/i9 *atf1*Δ/Δ) or (B) with *ATF1* (s52→i9) transformed with overexpression plasmids. Fermentations were carried out in YP250–10% glucose with 200 mg/liter hygromycin to maintain the plasmids. The table shows average aroma production values from four fermentation replicates ± SD and the significance of any deviation from the mean of the strains containing empty plasmid. Download TABLE S3, DOCX file, 0.1 MB. Copyright © 2018 Holt et al. 2018 Holt et al. This content is distributed under the terms of the Creative Commons Attribution 4.0 International license.

The overexpression results with the two different *EAT1* alleles also confirm their causative character for the difference in ethyl acetate production between the respective parent strains. In s52/i9 *atf1*Δ/Δ, *EAT1* overexpression also increased isobutyl and isoamyl acetate levels significantly, but they remained very low compared to those of a strain containing *ATF1* (see Table S4A and B in the supplemental material). This indicates that the Eat1 enzyme has low residual alcohol acetyl-CoA transferase activity with other alcohols besides ethanol. Isoamyl alcohol and isobutanol levels were also increased in the s52/i9 *atf1*Δ/Δ strain. Overexpression of *IMO32* also affected ethyl ester production, but only in the absence of *ATF1* (see Tables S3A and S4B in the supplemental material). *IMO32* did not cause any significant difference in the level of any of the flavor compounds in the presence of *ATF1* in the s52/i9 strain (Table S3B).

10.1128/mBio.01279-18.6 TABLE S4  Aroma compounds produced in fermentations of the engineered industrial Anchor NT112 and Kyokai no. 7 wine and saké yeast strains, as well as the MauriBrew Ale 514 beer strain, in industrial-like medium. Shown are aroma profiles produced in fermentations by (A) Anchor NT112, (B) Kyokai no. 7, and (C) MauriBrew Ale 514 strains engineered with *EAT1* frameshift and *SNF8* nonsense mutations. The strains were fermented in synthetic Chardonnay grape must (Anchor NT112), YP250–25% (wt/vol) glucose (Kyokai no. 7), or malt extract medium at 15°Brix (MauriBrew Ale 514), all at 18°C with 130 rpm magnetic stirring. The significance of any deviation from the mean of the control strain is indicated as follows: ns, not significant (*P* > 0.05); *, *P* ≤ 0.05; **, *P* ≤ 0.01; ***, *P* ≤ 0.001. All fermentations were carried out with four replicates, and aroma production values are shown ± SD. Download TABLE S4, DOCX file, 0.1 MB. Copyright © 2018 Holt et al. 2018 Holt et al. This content is distributed under the terms of the Creative Commons Attribution 4.0 International license.

A BLAST search with *EAT1* using all available sequences revealed only 109 genes with significant sequence similarity (E value of ≤10^−15^), while with *IMO32*, 5,026 genes with significant sequence similarity (E value of ≤10^−15^) were found. This shows that *EAT1* is much less distributed in nature compared to *IMO32*, although it is conserved in the *Saccharomyces* species, including the S. cerevisiae and Saccharomyces eubayanus paralogs in the S. pastorianus lager yeast. The discovery of *EAT1* highlights the use of pooled-segregant QTL analysis as an unbiased tool for identification of new metabolic enzymes, in particular in aroma production.

### Engineering of *EAT1* and *SNF8* in the strains Anchor NT112, Kyokai no. 7, and MauriBrew Ale 514.

To further evaluate the potential for improving industrial strains used in beverage production, we have engineered the new *EAT1* and *SNF8* variants in three diploid industrial strains using the CRISPR/Cas9 methodology. We chose the parental diploid Anchor NT112 wine yeast, from which the inferior haploid i9 spore was obtained for QTL analysis, and the Kyokai no. 7 type strain for saké fermentations, as well as the MauriBrew Ale 514 brewing strain, which are both unrelated to any of the strains used for QTL analysis.

**FIG 10:**
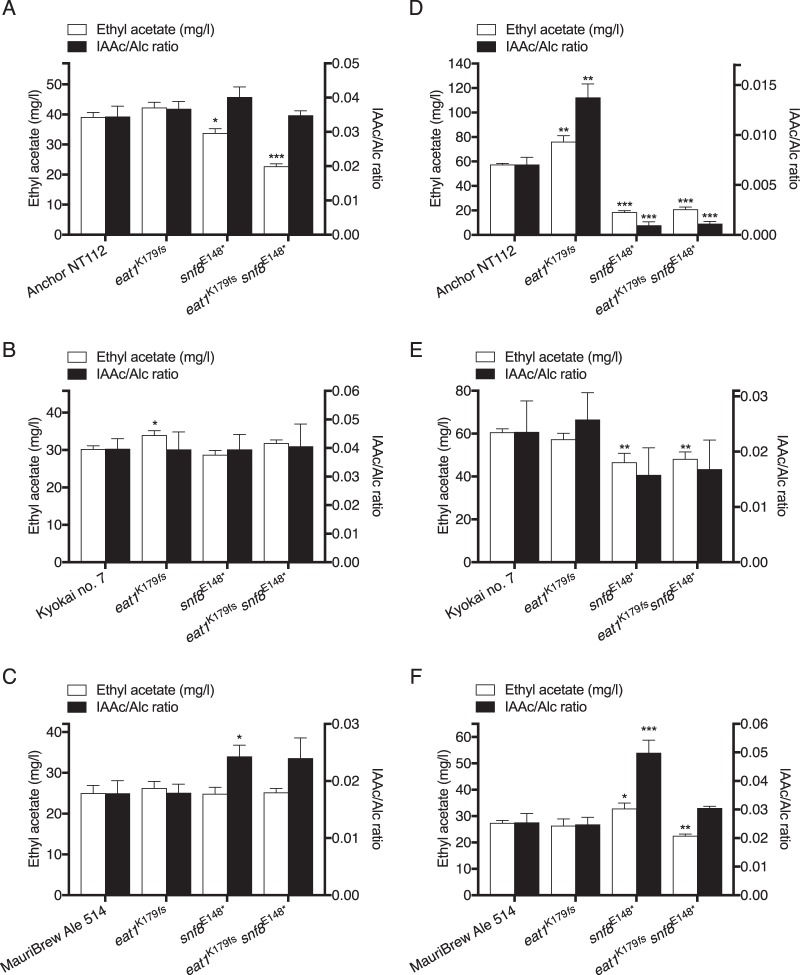
Evaluation of *EAT1* and *SNF8* SNPs in the industrial strains NT112 and Kyokai no. 7. The ethyl acetate production profile and isoamyl acetate/isoamyl alcohol ratio (isoamyl acetate productivity) of the engineered strains are shown. Fermentation was carried out with YP250–10% glucose for NT112 (A), Kyokai no. 7 (B), and MauriBrew Ale 514 (C), and with synthetic Chardonnay grape must (45) with 220 g/liter glucose plus fructose for NT112 (D), YP250–25% glucose for Kyokai no. 7 (E), and malt extract medium at 14.5°Plato for MauriBrew Ale 514 (F). Fermentations were inoculated with cultures grown overnight in YP250–2% glucose and carried out at 25°C for fermentations with YP250–10% glucose or 18°C for industrial-like conditions in 100 ml, stirred at 130 rpm. The complete aroma profiles are available in Table S4 and S5. Significance indications refer to the differences in production levels between the engineered strains and the respective unmodified strains.

First, we performed fermentations with the strains in YP250–10% glucose to evaluate the flavor metabolite production of strains in the same medium. Clear strain-specific differences were found for the three mutant industrial strains. For the strains with *eat1*^E179*fs*^, only ethyl decanoate was increased in NT112 (Fig. 10A; Table S4A), whereas ethyl acetate was slightly increased for Kyokai no. 7 (Fig. 10B), and isobutanol and isoamyl alcohol were significantly increased in MauriBrew Ale 514, which led to an increase in isobutyl and isoamyl acetate without affecting the IAAc/Alc ratio (Fig. 10C; Table S4C). Ethyl acetate was very significantly decreased by introducing the *snf8*^K148^* nonsense mutation in the NT112 wine strain without reducing the IAAc/Alc ratio (Fig. 10A), similarly to its haploid i9 derivative (Fig. 7C). Interestingly, introduction of *snf8*^K148^* led to a very significant increase in the IAAc/Alc ratio in the MauriBrew Ale 514 strain, without increasing ethyl acetate production, which suggests a significant increase in the *ATF1* AATase activity in this strain. The fermentation capacity was only slightly reduced for NT112 (see Fig. S2A in the supplemental material), similarly to its haploid i9 derivative (Fig. 7D), whereas a much stronger inhibitory effect was seen in Kyokai no. 7 and MauriBrew Ale 514 (Fig. S2B and C).

10.1128/mBio.01279-18.2 FIG S2  Fermentation profile of industrial yeast strains engineered with *eat1*^K179*fs*^ and *snf8*^E148^*. The fermentation progress derived from weight loss due to CO_2_ evaporation is shown for the engineered strains. Fermentation was carried out with YP250–10% glucose for NT112 (A), Kyokai no. 7 (B), and MauriBrew Ale 514 (C) and with synthetic Chardonnay grape must (45) with 220 g/liter glucose plus fructose for NT112 (D), YP250–25% glucose for Kyokai no. 7 (E), and malt extract medium at 14.5°Plato for MauriBrew Ale 514 (F). Download FIG S2, EPS file, 0.2 MB. Copyright © 2018 Holt et al. 2018 Holt et al. This content is distributed under the terms of the Creative Commons Attribution 4.0 International license.

To evaluate the potential of *eat1*^E179*fs*^ and *snf8*^K148^* for industrial strain improvement, we carried out fermentations in a medium as relevant as possible for the respective application of the three strains: 25% glucose YP medium adjusted to 250 mg/liter free amino nitrogen (FAN), synthetic Chardonnay grape must, and malt extract medium (wort). Engineering of *snf8*^E148^* lowered ethyl acetate significantly for both NT112 and Kyokai no. 7 in the relevant medium, but also decreased the isoamyl acetate productivity to a strain-dependent extent, with a severe reduction for NT112, whereas Kyokai no. 7 was much less affected (Fig. 10D and E). On the other hand, the effect of *snf8*^E148^* in MauriBrew Ale 514 beer fermentation was remarkably positive with a 2-fold increase of the IAAc/Alc ratio, but with a very limited increase in ethyl acetate (Fig. 10F). In fact, many of the positive fruity esters (i.e., isoamyl acetate, isobutyl acetate, ethyl octanoate, and ethyl decanoate) were all significantly enhanced without affecting the higher alcohols (see Table S5C in the supplemental material). This increase in the fruity esters was even more pronounced with fermentation in wort compared to YP250 synthetic medium. In addition, there was much less reduction in fermentation capacity for MauriBrew Ale 514 in malt extract medium compared to YP250–10% glucose, which may be due to the fact that maltose is the main sugar for fermentation in malt extract medium. Surprisingly, this result indicates a strong industrial potential for improving the IAAc/Alc ratio by introduction of the *snf8*^E148^* mutation in brewing yeast.

10.1128/mBio.01279-18.7 TABLE S5  Aroma compounds produced in fermentations of the engineered industrial Anchor NT112 wine and Kyokai no. 7 saké yeast strains, as well as the MauriBrew Ale 514 beer strain, in industrial-like medium. Shown are aroma profiles produced in fermentations by (A) Anchor NT112, (B) Kyokai no. 7, and (C) MauriBrew Ale 514 strains engineered with *EAT1* frameshift and *SNF8* nonsense mutations. The strains were all fermented with YP250–10% (wt/vol) glucose at 25°C with 130 rpm magnetic stirring. The significance of any deviation from the mean of the control strain is indicated as follows: ns, not significant (*P* > 0.05); *, *P* ≤ 0.05; **, *P* ≤ 0.01; ***, *P* ≤ 0.001. All fermentations were carried out with four replicates, and aroma production values are shown ± SD. Download TABLE S5, DOCX file, 0.1 MB. Copyright © 2018 Holt et al. 2018 Holt et al. This content is distributed under the terms of the Creative Commons Attribution 4.0 International license.

In contrast to fermentation with YP250–10%glucose, fermentation with synthetic Chardonnay grape must for the NT112 wine strain with the *eat1*^E179*fs*^ frameshift mutation gave a significant increase in acetate ester levels (including ethyl acetate), without altering higher alcohol levels, and resulted in a 2-fold-higher isoamyl acetate/alcohol ratio (Fig. 10D; Table S5A). Except for an increase in isobutanol and isobutyl acetate in MauriBrew Ale 514 (Table S5C), no difference in flavor production was found after introduction of *eat1*^E179*fs*^ in the Kyokai no. 7 or the MauriBrew Ale 514 backgrounds (Fig. 10E and F).

## DISCUSSION

Ethyl acetate is a common organic solvent used in paints, lacquers, nail polish removers, etc., with an estimated global production of 3.5 million tons per year in 2015 (30). Currently, it is produced by chemical synthesis, but biological production by yeast or bacteria provides a sustainable alternative that has gained increasing interest. In brewing of alcoholic beverages, on the other hand, ethyl acetate gives a solvent-like off-flavor, and it is therefore undesirable at concentrations above its aroma threshold. The existence of unknown flavor enzymes in ethyl acetate production has been known for 15 years, since a double *atf1Δ atf2Δ* strain still produces about 50% of the normal ethyl acetate level (6). In the present study, we have identified a new flavor-producing enzyme, Eat1, by using pooled-segregant QTL analysis. We show that a specific frameshift mutation in the *EAT1* gene strongly affects the level of ethyl acetate in alcoholic fermentations. Unexpectedly, however, the level of ethyl acetate increased when the *ATF1* WT gene was present and the *eat1*^K179*fs*^ mutation was introduced in the inferior i9 haploid. This was due to an increase in the *ATF1*-derived AATase activity as isoamyl acetate, which is uniquely produced by the Atf1 enzyme, was increased while the level of its precursor fusel alcohol, isoamyl alcohol, remained the same (Fig. 8). Our results show that the Eat1 and Atf1 enzymes are intertwined, with Atf1 partially overriding the Eat1 effect on ethyl acetate. The most likely explanation appears to be that they have unknown overlapping cellular functions. It is therefore also possible that the deletion of *ATF1* may induce the activity of the Eat1 enzyme and lead to overestimation of the remaining ethyl acetate production.

Correct cellular localization is often required for proper functioning of enzymes in metabolic pathways. However, the reasons for targeting of an enzyme to a specific cellular compartment are sometimes unclear, and their cellular enzymatic function may even be restored by expression without the innate targeting signal. This was explored for bio-based production of isoamyl and isobutyl acetate esters with yeast, by engineering enhanced flux through the mitochondrial isoleucine-leucine-valine pathway, together with mitochondrial expression of the Atf1 enzyme. However, mitochondrial expression of Atf1 resulted in lower levels of acetate esters compared to cytosolic expression in the engineered strain (31). The Atf1 protein contains two hydrophobic stretches of amino acids that make it membrane associated in lipid particles (32). Fluorescently tagged Eat1 has been found localized to the mitochondria in high-throughput screening (33), which is consistent with its predicted N-terminal targeting signal peptide. It is possible that yeasts with mutations in the Eat1 mitochondrial targeting signal causing a higher level of cytosolic protein will produce an altered flavor ester profile.

The *ATF1* gene has been shown to function in dispersal of yeast by fruit flies, particularly due to its production of ethyl acetate (34). As Eat1 also produces ethyl acetate, it may also be important for attraction of fruit flies. However, as is often the case for flavor-producing enzymes, Atf1 and Eat1 may have other biological functions besides flavor production and could potentially acetylate or hydrolyze unknown substrates in other parts of cellular metabolism. Overexpression of the *EAT1* gene caused a significant increase in ethyl acetate and a modest increase in isobutyl and isoamyl acetate in the absence of *ATF1* (Table S3A and B). These results therefore suggest that the substrate specificity for Eat1 is primarily limited to ethanol with some residual activity toward other alcohols. This is in stark contrast to Atf1, which is remarkably promiscuous for alcohol substrates (6, 35, 36). Atf1, as well as the ethanol acyl-CoA transferases Eht1 and Eeb1, also shows significant *in vitro* thioesterase activity (8, 37, 38), with Atf1 having the capacity to hydrolyze long-chain fatty acid acyl-CoAs (obeying Michaelis-Menten kinetics up to dodecanoyl-CoA, C_12_) (37). Future experiments should clarify whether Eat1, Atf1, Eht1, and Eeb1, are also involved in fatty acid and coenzyme A homeostasis or if they could acetylate nonvolatile and potentially biologically active long-chain alcohols and fatty acids.

Interestingly, restoration of the full-length gene in s52 (SNF8*^148E^) caused a very significant increase in production of fusel alcohols. On the other hand, acetaldehyde was only increased in i9 upon expression of truncated *SNF8* (*snf8*^E148^*). The increased level of fusel alcohols may suggest that *SNF8* plays a role in regulation of the NADH/NAD^+^ redox balance, *de novo* synthesis of branched-chain amino acids, and/or regulation of the enzymes in the Ehrlich pathway. Mutants in the *SNF8* gene were first isolated based on strongly reduced growth on raffinose. However, we observed only a modest growth reduction of the i9 *snf8*^E148^* strain on raffinose, in which it was mainly affected in the lag phase of growth and onset of fermentation (Fig. 9). The molecular function of Snf8 (also known as Vps22) has been intensively studied as a member of the endosomal complex required for targeting (ESCRT II), which together with ESCRT I and III facilitates endocytosis and protein sorting to the vacuole (39–41). Snf8 (as well as other members of ESCRT II) is required for endosomal function, and null mutants have impaired formation of multivesicular bodies and form large endosome structures with stalled cargo (42). It therefore seems likely that Snf8 is indirectly linked (e.g., by endocytosis of a protein from the cell membrane or vacuole) to a system that regulates both flavor production and derepression of the *SUC2* invertase gene.

In this study, selection was performed for both high and low production of ethyl acetate, and to our surprise, we observed a lack of overlapping QTL in these two pools of segregants, in both the presence and absence of *ATF1* (except for QTL1 and QTL2). Fujiwara et al. (43) reported that a null mutation of the *SCH9* protein kinase gene lowers the expression level of *ATF1*. The *SCH9* gene is situated in the middle of the homozygous region at the right arm of chromosome eight (Fig. 5) and contains a nonsynonymous SNP, *SCH9*^E430K^, which we have not investigated further. Although we find it unlikely, given that the segregation of ethyl acetate production in the s52/i9 WT *ATF1* offspring was within the range of the haploid parent strains, we cannot rule out that this region (131 kbp without considering any possibly unmapped subtelomeric regions) could potentially cause a difference in levels of ethyl acetate production between the *atf1*Δ and WT *ATF1* backgrounds.

Although many causative alleles for industrially important traits have been identified, very little work has been reported on the systematic introduction of such alleles in other genetic backgrounds to evaluate their general usefulness for targeted industrial strain improvement. We have engineered the *eat1*^K179*fs*^ and *snf8*^E148^* mutations in the Anchor NT112 wine yeast, the Kyokai no. 7 saké strain, and the MauriBrew Ale 514 brewing strain. This caused the expected reduction in ethyl acetate production in the former two backgrounds but not in the latter strain. The reduction in ethyl acetate production in the former two strains was correlated with a strong and undesirable reduction in the IAAc/Alc ratio. However, in the Ale 514 brewing strain, the reverse effect was observed. Ethyl acete production was slightly increased, while the IAAc/Alc ratio was strongly enhanced without any effect on the isoamyl alcohol level. Also the level of the highly desirable fruity ethyl esters ethyl octanoate and ethyl decanoate was strongly increased. This could improve the fruitiness of the beer aroma profile without giving an undesirable vinous higher-alcohol off-flavor and thus represent a desirable outcome for brewing yeast strain improvement. There did not appear to be significant effects on other traits important for industrial application, although only follow-up evaluation at pilot and commercial scales can give final confirmation in this respect. Establishment of a large portfolio of causative alleles for multiple commercially important traits could thus provide a highly effective tool for targeted strain improvement of industrial yeast strains.

In conclusion, we have identified the genes *EAT1* and *SNF8* by performing pooled-segregant QTL analysis of ethyl acetate production in a strain lacking *ATF1*, encoding the major broad-range acetate ester biosynthesis enzyme. The *EAT1* gene encodes a novel enzyme that specifically produces ethyl acetate, which makes it an excellent target for abolishing solvent-like off-flavor in alcoholic beverages produced by yeast fermentation. We found that most of the QTLs identified were not correlated for low and high production of ethyl acetate, indicating that epistasis occurs and that the genetics of acetate ester production is also highly complex, as is generally thought to be the case for secondary flavor and aroma metabolism. This is also consistent with its high variability under different fermentation conditions (2). In addition, we have engineered the causative *EAT1* and *SNF8* mutations in three industrial yeast strains, which showed their potential to improve flavor production, but in a strain background-dependent manner. This illustrates the necessity of introducing variants in different genetic backgrounds in order to assess their general potency and shows that the fermentation medium is critical for determination of industrial applicability, highlighted by the potential of the *snf8*^E148^* mutation for engineering flavor metabolism in brewing yeast.

## MATERIALS AND METHODS

### S. cerevisiae strains.

The S. cerevisiae strains used in this work are shown in Table S6 in the supplemental material.

10.1128/mBio.01279-18.8 TABLE S6  Strains used in this study. Download TABLE S6, DOCX file, 0.1 MB. Copyright © 2018 Holt et al. 2018 Holt et al. This content is distributed under the terms of the Creative Commons Attribution 4.0 International license.

### Chemicals.

All flavor compounds, solvents, and components of media were obtained from Sigma-Aldrich or Merck unless otherwise mentioned.

### Fermentation conditions.

Fermentations were carried out at 25°C with gentle magnetic stirring at 130 rpm in 100-ml fermentation tubes, validated against European Brewing Convention (EBC) 2-liter tall tubes, fitted with water locks to create semianaerobic conditions. The same pitching rate of 0.5 to 1.0 million cells/ml, as in wine production, was used (equivalent to an optical density at 600 nm [OD_600_] of about 0.07). To mimic industrial fermentation conditions as closely as possible, we used 0.27% (wt/vol) yeast extract (Merck) and 0.54% (wt/vol) bacteriologic peptone, which results in a FAN level of about 250 mg/liter (YP250) (44, 45). This was based on information from the suppliers mentioning α-amino nitrogen levels of 26 mg/g in yeast extract and 40 mg/g in bacteriologic peptone. The FAN level was confirmed in two different batches (212.2 and 256.4 mg/liter) by the EBC 9.10 spectrophotometric method, which measures ammonia, amino acids, and terminal α-amino nitrogen of peptides and proteins. The pH was lowered to 4.5 with concentrated hydrochloric acid. The low inoculation rate used allowed us to inoculate 100 ml of fermentation medium (YP250–10% glucose) with an aliquot of 0.5 ml from cultures grown overnight in YP250–2% glucose. Weight loss of the fermentation tubes due to CO_2_ release was used to estimate fermentation progress.

### Wine, saké, and beer fermentations for Anchor NT112, Kyokai no. 7, and MauriBrew Ale 514.

We used media mimicking wine, saké, or beer brewing to evaluate the effect of engineering the superior SNPs in *EAT1* and *SNF8* in the industrial strains. For the Anchor NT112 wine yeast, synthetic Chardonnay grape must, according to values based on a large characterization of Chardonnay grapes in Australia (45), was composed from chemicals obtained from Sigma-Aldrich. Separate stocks of amino acids (dissolved in H_2_O from a mixture of amino acids, pH 2.5), trace elements (kept at −20°C), and vitamins (kept at −20°C), together with salts, acids, and sugars, were added stepwise under stirring to ensure solubility of all the components. After adjusting the final pH to 3.5 with HCl/KOH, the solution was filter sterilized through a 0.2-µm-pore filter, and lipids (kept at −20°C in absolute ethanol) and sterols (β-sitosterol [kept at −20°C] in absolute ethanol) were added directly. For the Kyokai no. 7 saké yeast, we used YP250 (as previously described) with 250 g/liter of glucose. Finally, for the MauriBrew Ale 514 brewing yeast, we used malt extract medium consisting of 166 g/liter of malt extract (Brewferm spraymalt 8 EBC; Brouwland, Belgium) supplemented with 0.5 mg/liter ZnSO_4_ and autoclaved at 110°C for 15 min. The final gravity was 14.5°Plato. After autoclaving, the malt extract medium was cold settled overnight and filtered through a nylon filter (GE Healthcare) to remove insoluble precipitates. The medium was subsequently purged with sterile pure O_2_ to obtain a dissolved O_2_ level of 20.4 mg/liter.

Inoculation was performed from overnight cultures in YP250–2%glucose for wine and saké fermentations at a final OD of 0.2 (roughly 2 × 10^6^ cells/ml). For beer fermentations, an overnight preculture was first made in YPD, reinoculated into malt extract medium, and allowed to grow for 48 h. The final inoculation rate was 9 × 10^7^ cells/ml (OD of 1.89).

All fermentations were incubated at 18°C with 130-rpm magnetic stirring with a medium volume of 100 ml in tall tubes equipped with water locks to ensure semianaerobic conditions.

### Screening of MCB strain collection.

The yeast strain collection used in this study included 104 natural isolates associated with human activity, such as wine production, beer brewing, cider making, and cheese production, 82 industrial wine strains, 42 natural isolates not associated with human activity (soil, bark of trees, etc.), 38 Ale brewing strains, 21 lager brewing yeasts, 32 brewing strains from various sources, 23 bioethanol strains, 23 Baker’s yeasts, 22 saké yeasts, and 8 strains used for spirit production, such as whiskey and vodka. Fifty strains were not able to grow efficiently and were unable to complete the fermentation (total weight loss, <4.5 g/100 ml) within 4 days. They were discarded from the analysis, leaving a final total of 429 strains. Their fermentations were subjected to quantification of aroma metabolites by gas chromatography coupled with a flame ionization detector (see Data S1 in the supplemental material).

10.1128/mBio.01279-18.9 DATA SET S1  Aroma profiles of strains in a subset of the MCB collection. Download DATA SET S1, XLSX file, 0.1 MB. Copyright © 2018 Holt et al. 2018 Holt et al. This content is distributed under the terms of the Creative Commons Attribution 4.0 International license.

The bioethanol production strain Ethanol Red was used as a general reference strain for determination of flavor production and was included in all 11 batches of strains evaluated. It produced 30.1 ± 3.8 mg liter^−1^ ethyl acetate, 3.3 ± 0.7 mg liter^−1^ isoamyl acetate, 0.34 ± 0.10 mg liter^−1^ isobutyl acetate, 0.52 ± 0.13 mg liter^−1^ ethyl hexanoate, 45.3 ± 7.6 mg liter^−1^ isobutanol, and 124.8 ± 15.3 mg liter^−1^ isoamyl alcohol (Data Set S1).

### Headspace gas chromatography.

Fermentation samples with a volume of 2 ml were analyzed in 15-ml vials with a Thermoscience Trace gas chromatograph equipped with a ResTek Stabilowax polyethylene glycol column with a 0.25-µm diameter. Split injection (1:25), with a resting flow rate of 2 ml/min of He, was performed after 10 min of equilibration at 60°C with a Thermoscience TriPlus RSH autosampler. The oven was kept at 40°C for 2 min, heated to 240°C with a ramping of 15°C/min, and kept at 240°C for 2 min. The detection was carried out with a flame ionization detector (FID), using 20 ml/min of N_2_, 350 ml/min high-grade compressed air, and 30 ml/min H_2_ provided from a VWR H_2_ generator. For the industrial strains, quantification was done under identical chromatography conditions, except that detection was performed with a Thermo Scientific ISQ single quadrupole mass spectrometer. Concentrated standards were kept at −20°C in gas chromatography (GC)-grade absolute ethanol and brought to the final concentration in 5% ethanol in volumetric dilutions, except for wine and saké fermentations, for which 15% ethanol was used in the standards. Fermentation samples were kept at 4°C prior to analysis.

### Determination of cellular DNA content.

Freshly grown cells on YPD agar were inoculated into liquid YPD and allowed to grow for 6 h. Exponentially growing cells were harvested after 6 h by centrifugation and kept on ice until the treatment for dissolution of cell aggregates. The aggregation observed was not due to typical flocculation, since addition of EDTA and mannose (both up to 250 mM) did not resolve the clumps of aggregated cells. Addition of the protease papain (at 10 U/ml) only caused partial dissolution of the flocs. The final protocol used a cell sonication step for 20 s on ice, followed by a gentle spin down at 500 g for 5 min. Cells were subsequently stained with propidium iodide, and the DNA content was measured with flow cytometry.

### Transformation of yeast.

The industrial isolate TMB 3000 and its segregants used in this study showed very poor transformation efficiency with standard transformation protocols. Hence, electroporation was used according to Benatuil et al. (46). Briefly, 1 ml of overnight-pregrown (aggregating) cell cultures was inoculated into 100 ml YPD and incubated for 3 h to obtain exponentially growing cells. The cells were then resuspended in 25 ml Tris-EDTA (TE) buffer (pH 8.0) with 0.1 M LiAc and 10 mM dithiothreitol (DTT) (freshly prepared) and incubated with gentle rotation for 1 h at 25°C. The pretreated cells were kept on ice, washed 2 times with 25 ml demineralized water (demi water), and once with 10 ml 1 M d-sorbitol. Cell pellets were then resuspended in 200 to 400 µl 1 M d-sorbitol. The final electroporation was performed with 40 µl cell suspension, 10 µl single-stranded salmon sperm DNA, and 10 µl DNA solution containing ≥500 ng DNA, with twice as much when two DNA fragments were cotransformed. The electroporation was carried out using a voltage of 1,500 kV. The selection based on antibiotic resistance was optimized, using final concentrations of 375 mg/liter geneticin, 20 mg/liter phleomycin, 200 mg/liter nourseothricin, and 300 mg/liter hygromycin for all transformations of Anchor NT112 and TMB 3000 and their segregants. For transformations of Kyokai no. 7 and MauriBrew Ale 514, the final concentrations of geneticin were 500 and 200 mg/liter, respectively, and those of hygromycin were 1,500 and 300 mg/liter, respectively. All genes were deleted by replacing the open reading frame with a split resistance marker fused with 400- to 500-bp flanking regions to facilitate homologous recombination.

### Construction of hybrids with TMB 3000 and NT112 and isolation of segregants.

To obtain heterothallic versions of the diploid strains TMB 3000 and NT112, the two copies of the HO endonuclease gene were deleted using the KanMX and BleMX selectable markers flanked by ϕC31 integrase attB and attP recognition sites. Subsequent mass sporulation was performed and diethyl ether treatment was used to kill the vegetative cells (47). The fermentation performance and aroma profile of the segregants of heterothallic TMB 3000 and NT112 were then assessed in small tube fermentations. Further selection was based on high or low ethyl acetate production and a high or low isoamyl acetate/ethyl acetate ratio. The selected segregants were transformed with a plasmid containing the ϕC31 intregrase gene under a galactose-inducible promoter to remove the KanMX and BleMX selectable markers on YP–2% galactose medium containing nourseothricin. This was done to recycle the selectable markers and avoid homologous recombination into the *HO* locus. The *ATF1* gene was deleted with the same resistance markers used for *HO* deletion. For deletion of *ATF2*, the HphMX marker was used.

To construct the hybrids used for QTL analysis, the s52 *ho*::BleMX and i9 *ho*::KanMX strains or the s52 *ho*::*atf1*::BleMX and i9 *ho*::*atf1*::KanMX strains were allowed to mate on YPD medium for 4 h before double selection was carried out with geneticin and phleomycin to select the diploid hybrids. These hybrids were then mass sporulated as previously described, growth of the segregants on medium with either geneticin or phleomycin was confirmed, and their mating type was determined by PCR.

### DNA isolation from segregants and identification of QTL.

Haploid segregants were isolated after sporulation of the s52/i9 *atf1*Δ and s52/i9 WT *ATF1* hybrid strains, and subjected to small tube fermentations to assess fermentation performance and the aroma profile. After 7 days of fermentation, the medium still contained more than 2 g/liter of residual glucose in fermentations with 66 (out of 573) *atf1*Δ segregants and 86 (out of 799) WT *ATF1* segregants, which were discarded from the analysis. The aroma metabolites were quantified by GC-FID in the remaining 507 *atf1*Δ and 713 WT *ATF1* segregants (see Data Set S2 in the supplemental material). Segregants showing a low (≤4 mg/liter for *atf1*Δ or ≤20 mg/liter for WT *ATF1* segregants), high (≥10 mg/liter for *atf1*Δ or ≥50 mg/liter for WT *ATF1* segregants), or random production of ethyl acetate were selected for pooled-segregant QTL analysis. Each pool of *atf1*Δ segregants consisted of 35 strains, and each pool of WT *ATF1* segregants consisted of 30 strains. Genomic DNA from each segregant was isolated with a phenol chloroform-isoamyl alcohol (PCI) method, which included proteinase K and RNase treatment. The DNA was quantified with the PicoGreen double-stranded DNA (dsDNA) assay kit (Invitrogen) and pooled in equal amounts. The DNA pools were sequenced at ~×70 coverage by Illumina HiSeq 2000 technology at BGI (Hong Kong). The genomes of the haploid s52 and i9 strains were sequenced at ~×30 coverage. Adapter trimming and quality control of the reads was performed by BGI and confirmed with FastQC.

10.1128/mBio.01279-18.10 DATA SET S2  Aroma profiles of segregants used in this study. Download DATA SET S2, XLSX file, 0.1 MB. Copyright © 2018 Holt et al. 2018 Holt et al. This content is distributed under the terms of the Creative Commons Attribution 4.0 International license.

Mapping of the Illumina reads was performed with Bowtie 2.0 (using the very sensitive flag option). SNP variant calling and filtering was performed with the Next Generation Sequencing Experience Platform (NGSEP) (48). A minimum coverage of 10× for all genomes and minimum distance between SNP variants of 50 bp was used for linkage mapping to minimize mapping and sequencing errors. EXPLoRA (49) was then used to extract biallelic SNPs and linkage probabilities (LOD scores) and 1-LOD drop intervals were calculated with MULTIPOOL (50) using 100-bp bin sizes.

### CRISPR/Cas9 modification of *EAT1* and *SNF8*.

Standard CRISPR/Cas9 technology was used (51). The guide RNA (gRNA) plasmid and the Cas9 expression plasmid were as described by DiCarlo et al. (52), with the auxotrophic marker changed for the antibiotic resistance marker as indicated and the cloning site modified for Gibson assembly. A single-copy Cas9 constitutive expression plasmid with a geneticin marker (KanMX) was first introduced by transformation. This strain was then transformed with a PCR-amplified oligonucleotide, as described below, together with gRNAs cloned into a multicopy plasmid containing the hygromycin resistance marker. The Cas9 and guide RNA plasmids were lost by culturing the strains without selection in three subsequent transfers (300-fold dilutions) in liquid media and confirming loss of the geneticin and hygromycin resistance markers. During the genetic modifications, we observed that the transformants of i9 acquired a tendency for loss of mitochondrial function as ~40 to 90% were unable to grow with nonfermentable carbon sources and showed smaller colony sizes (formation of petites). We therefore isolated i9 clones and transformants which could grow with glycerol as the sole carbon source and only used cells freshly cultured on glycerol for all experiments.

The *EAT1* 532delA and 531_insA_533 indel mutations in s52 and i9, respectively, were swapped into the *EAT1* locus with the following guide RNA: 5′-AGAGATGTCAAGATTTTAAG and a 157-bp donor DNA oligonucleotide containing the frameshift mutation and a synonymous A510G mutation (codon change in leucine TTU to TTG). The synonymous mutation was introduced within proximity of the target mutation since the *EAT1* open reading frame did not contain a suitable mutation close to a Cas9 protospacer adjacent motif (PAM) site for correct targeting. For *SNF8*, the G442T nonsense mutation was targeted in s52 with the guide RNA 5′-GAAATATCTAAAAACACTCT and in i9 with 5′-GAAATATCTCAAAACACTCT. The donor DNA was a 601-bp oligonucleotide amplified from genomic DNA and containing only the G442T mutation.

Engineering of NT112, Kyokai no. 7, and MauriBrew Ale 514 was performed as described above for the i9 strain, with the following exceptions. The *SNF8* gene in Kyokai no. 7 was targeted with the guide RNA 5′-GAAATATCTCAAAACATTCT and was provided as an 80-bp duplex oligonucleotide only containing the G442T mutation, since it contained a mutation in the seed sequence (G435A). The correct integration of the *EAT1* mutation in both alleles of the diploid strains was confirmed by Sanger sequencing. The success rates for integration of the synonymous mutation and the indel (22 bp upstream) in both alleles were 9% (1/11), 33% (4/12), and 0% (0/59) for NT112, Kyokai no. 7, and MauriBrew Ale 514, respectively. Because of the very low success rate for MauriBrew Ale 514, we introduced another synonymous SNP (codon change in serine TCC→TCT [C519T]) closer to the indel (11 bp upstream). This synonymous SNP is also naturally present in the *EAT1* gene of the W34/70 type lager yeast. It was inserted into the genome together with the indel using the guide RNA 5′-TTTTAAGAGGTTCCCCCAGC.

*SNF8* contained the same synonymous SNP as in Kyokai no. 7 (G435A), but was heterozygous for this mutation (435G/A). Therefore, we first targeted with the gRNA prepared for Kyokai no. 7 (435A), to make the synonymous SNP homozygous, and subsequently targeted with the gRNA used for i9 (435G). These modifications allowed efficient CRISPR/Cas editing of both *EAT1* and *SNF8* in the diploid industrial strains.

The gRNAs were designed for S. cerevisiae with the DNA 2.0 CRISPR gRNA design tool and checked for potential off-targeting with BLAST analysis. The efficiency of the gRNA was evaluated *in silico* with the CRISPR Efficiency Predictor (http://www.flyrnai.org/evaluateCrispr/).

### Overexpression of *EAT1* and *IMO32*.

The alleles of *EAT1* and *IMO32* from the i9 and s52 strains were cloned into the high-copy p426 plasmid containing a yeast 2µm replication origin and a hygromycin-selectable marker (hphMX). The open reading frames were cloned in-frame with the TEF1 promoter and terminator using BamHI and XhoI restriction sites for *EAT1* and SpeI and XhoI restriction sites for *IMO32*, respectively. Hygromycin in a concentration of 200 mg/liter was added in the YP250–10% glucose fermentation medium to maintain the plasmid during fermentation.

### Statistical analysis.

To determine the significance of the results, *t* tests with Holm-Sidak correction for multiple testing without assumption of consistent standard deviation were applied. Asterisks indicate *P* values as follows: *, *P* ≤ 0.05; **, *P* ≤ 0.01; and ***, *P* ≤ 0.001. “NS” indicates nonsignificant (*P* > 0.05). All experiments for which significance is indicated were carried out in quadruplicate.

### Data availability.

The Illumina genome sequencing data of the s52 and i9 haploid segregants and the superior, inferior, and unselected pools obtained from the hybrids s52/i9 *ATF1*/*ATF1* and s52/i9 *atf1*Δ/*atf1*Δ, respectively, will be available in the SRA database with the identifier SRP107919 (BioProject no. PRJNA387678).

## References

[1] Cordente AG, Curtin CD, Varela C, Pretorius IS 2012 Flavour-active wine yeasts. Appl Microbiol Biotechnol 96:601–618. doi:10.1007/s00253-012-4370-z. 22940803 PMC3466427

[2] Verstrepen KJ, Derdelinckx G, Dufour JP, Winderickx J, Thevelein JM, Pretorius IS, Delvaux FR 2003 Flavor-active esters: adding fruitiness to beer. J Biosci Bioeng 96:110–118. doi:10.1016/S1389-1723(03)90112-5. 16233495

[3] Fujii T, Nagasawa N, Iwamatsu A, Bogaki T, Tamai Y, Hamachi M 1994 Molecular cloning, sequence analysis, and expression of the yeast alcohol acetyltransferase gene. Appl Environ Microbiol 60:2786–2792. 8085822 10.1128/aem.60.8.2786-2792.1994 PMC201724

[4] Fujii T, Yoshimoto H, Tamai Y 1996 Acetate ester production by *Saccharomyces cerevisiae* lacking the *ATF1* gene encoding the alcohol acetyltransferase. J Ferment Bioeng 81:538–542. doi:10.1016/0922-338X(96)81476-0.

[5] Nagasawa N, Bogaki T, Iwamatsu A, Hamachi M, Kumagai C 1998 Cloning and nucleotide sequence of the alcohol acetyltransferase II gene (*ATF2*) from Saccharomyces cerevisiae Kyokai no. 7. Biosci Biotechnol Biochem 62:1852–1857. doi:10.1271/bbb.62.1852. 9836419

[6] Verstrepen KJ, Van Laere SD, Vanderhaegen BM, Derdelinckx G, Dufour JP, Pretorius IS, Winderickx J, Thevelein JM, Delvaux FR 2003 Expression levels of the yeast alcohol acetyltransferase genes *ATF1*, *Lg*-*ATF1*, and *ATF2* control the formation of a broad range of volatile esters. Appl Environ Microbiol 69:5228–5237. doi:10.1128/AEM.69.9.5228-5237.2003. 12957907 PMC194970

[7] Kruis AJ, Levisson M, Mars AE, van der Ploeg M, Garcés Daza F, Ellena V, Kengen SWM, van der Oost J, Weusthuis RA 2017 Ethyl acetate production by the elusive alcohol acetyltransferase from yeast. Metab Eng 41:92–101. doi:10.1016/j.ymben.2017.03.004. 28356220

[8] Saerens SM, Verstrepen KJ, Van Laere SD, Voet AR, Van Dijck P, Delvaux FR, Thevelein JM 2006 The *Saccharomyces cerevisiae EHT1* and *EEB1* genes encode novel enzymes with medium-chain fatty acid ethyl ester synthesis and hydrolysis capacity. J Biol Chem 281:4446–4456. doi:10.1074/jbc.M512028200. 16361250

[9] Den Abt TD, Souffriau B, Foulquié-Moreno MR, Duitama J, Thevelein JM 2016 Genomic saturation mutagenesis and polygenic analysis identify novel yeast genes affecting ethyl acetate production, a non-selectable polygenic trait. Microb Cell 3:159–175. doi:10.15698/mic2016.04.491. 28357348 PMC5349090

[10] Harington A, Herbert CJ, Tung B, Getz GS, Slonimski PP 1993 Identification of a new nuclear gene (*CEM1*) encoding a protein homologous to a beta-keto-acyl synthase which is essential for mitochondrial respiration in *Saccharomyces cerevisiae*. Mol Microbiol 9:545–555. doi:10.1111/j.1365-2958.1993.tb01715.x. 8412701

[11] Parts L 2014 Genome-wide mapping of cellular traits using yeast. Yeast 31:197–205. doi:10.1002/yea.3010. 24700360

[12] Pais TM, Foulquié-Moreno MR, Thevelein JM 2014 QTL mapping by pooled-segregant whole-genome sequencing in yeast. Methods Mol Biol 1152:251–266. doi:10.1007/978-1-4939-0563-8_15. 24744038

[13] Breunig JS, Hackett SR, Rabinowitz JD, Kruglyak L 2014 Genetic basis of metabolome variation in yeast. PLoS Genet 10:e1004142. doi:10.1371/journal.pgen.1004142. 24603560 PMC3945093

[14] Hubmann G, Mathé L, Foulquié-Moreno MR, Duitama J, Nevoigt E, Thevelein JM 2013 Identification of multiple interacting alleles conferring low glycerol and high ethanol yield in Saccharomyces cerevisiae ethanolic fermentation. Biotechnol Biofuels 6:87. doi:10.1186/1754-6834-6-87. 23759206 PMC3687583

[15] Marullo P, Aigle M, Bely M, Masneuf-Pomarède I, Durrens P, Dubourdieu D, Yvert G 2007 Single QTL mapping and nucleotide-level resolution of a physiologic trait in wine *Saccharomyces cerevisiae* strains. FEMS Yeast Res 7:941–952. doi:10.1111/j.1567-1364.2007.00252.x. 17537182

[16] Noble J, Sanchez I, Blondin B 2015 Identification of new *Saccharomyces cerevisiae* variants of the *MET2* and *SKP2* genes controlling the sulfur assimilation pathway and the production of undesirable sulfur compounds during alcoholic fermentation. Microb Cell Fact 14:68. doi:10.1186/s12934-015-0245-1. 25947166 PMC4432976

[17] Trindade de Carvalho B, Holt S, Souffriau B, Lopes Brandão R, Foulquié-Moreno MR, Thevelein JM 2017 Identification of novel alleles conferring superior production of rose flavor phenylethyl acetate using polygenic analysis in yeast. mBio 8:e01173-17. doi:10.1128/mBio.01173-17. 29114020 PMC5676035

[18] Steyer D, Ambroset C, Brion C, Claudel P, Delobel P, Sanchez I, Erny C, Blondin B, Karst F, Legras JL 2012 QTL mapping of the production of wine aroma compounds by yeast. BMC Genomics 13:573. doi:10.1186/1471-2164-13-573. 23110365 PMC3575298

[19] Eder M, Sanchez I, Brice C, Camarasa C, Legras JL, Dequin S 2018 QTL mapping of volatile compound production in Saccharomyces cerevisiae during alcoholic fermentation. BMC Genomics 19:166. doi:10.1186/s12864-018-4562-8. 29490607 PMC5831830

[20] Roncoroni M, Santiago M, Hooks DO, Moroney S, Harsch MJ, Lee SA, Richards KD, Nicolau L, Gardner RC 2011 The yeast *IRC7* gene encodes a beta-lyase responsible for production of the varietal thiol 4-mercapto-4-methylpentan-2-one in wine. Food Microbiol 28:926–935. doi:10.1016/j.fm.2011.01.002. 21569935

[21] Verstrepen KJ, Moonjai N, Bauer FF, Derdelinckx G, Dufour JP, Winderickx J, Thevelein JM, Pretorius IS, Delvaux FR 2003 Genetic regulation of ester synthesis in yeast: new facts, insights and implications for the brewer, p 234–248. *In* Smart K (ed), Brewing yeast fermentation performance, 2nd ed. Wiley-Blackwell, Oxford, United Kingdom.

[22] Verstrepen KJ, Derdelinckx G, Dufour JP, Winderickx J, Pretorius IS, Thevelein JM, Delvaux FR 2003 The alcohol acetyltransferase gene is a target of the cAMP/PKA and FGM nutrient-signalling pathways. FEMS Yeast Res 4:285–296. doi:10.1016/S1567-1356(03)00166-1. 14654433

[23] Lilly M, Lambrechts MG, Pretorius IS 2000 Effect of increased yeast alcohol acetyltransferase activity on flavor profiles of wine and distillates. Appl Environ Microbiol 66:744–753. doi:10.1128/AEM.66.2.744-753.2000. 10653746 PMC91891

[24] Steensels J, Meersman E, Snoek T, Saels V, Verstrepen KJ 2014 Large-scale selection and breeding to generate industrial yeasts with superior aroma production. Appl Environ Microbiol 80:6965–6975. doi:10.1128/AEM.02235-14. 25192996 PMC4249010

[25] Lindén T, Peetre J, Hahn-Hägerdal B 1992 Isolation and characterization of acetic acid-tolerant galactose-fermenting strains of *Saccharomyces cerevisiae* from a spent sulfite liquor fermentation plant. Appl Environ Microbiol 58:1661–1669. 1622236 10.1128/aem.58.5.1661-1669.1992 PMC195655

[26] Meilgaard MC 1982 Prediction of flavor differences between beers from their chemical composition. J Agric Food Chem 30:1009–1017. doi:10.1021/jf00114a002.

[27] Vallier LG, Carlson M 1991 New *SNF* genes, *GAL11* and *GRR1* affect *SUC2* expression in *Saccharomyces cerevisiae*. Genetics 129:675–684. 1752413 10.1093/genetics/129.3.675 PMC1204735

[28] Teo H, Perisic O, González B, Williams RL 2004 ESCRT-II, an endosome-associated complex required for protein sorting: crystal structure and interactions with ESCRT-III and membranes. Dev Cell 7:559–569. doi:10.1016/j.devcel.2004.09.003. 15469844

[29] Yeghiayan P, Tu J, Vallier LG, Carlson M 1995 Molecular analysis of the *SNF8* gene of *Saccharomyces cerevisiae*. Yeast 11:219–224. doi:10.1002/yea.320110304. 7785322

[30] Market Publishers, Ltd 2014 Global ETAC production to exceed 3.5 Mln tonnes in 2015, according to new report by Merchant Research and Consulting. PRWeb, Beltsville, MD http://prweb.com/releases/2014/02/prweb11619424.htm.

[31] Yuan J, Mishra P, Ching CB 2016 Metabolically engineered *Saccharomyces cerevisiae* for branched-chain ester productions. J Biotechnol 239:90–97. doi:10.1016/j.jbiotec.2016.10.013. 27746307

[32] Verstrepen KJ, Van Laere SD, Vercammen J, Derdelinckx G, Dufour JP, Pretorius IS, Winderickx J, Thevelein JM, Delvaux FR 2004 The *Saccharomyces cerevisiae* alcohol acetyl transferase Atf1p is localized in lipid particles. Yeast 21:367–377. doi:10.1002/yea.1100. 15042596

[33] Huh WK, Falvo JV, Gerke LC, Carroll AS, Howson RW, Weissman JS, O’Shea EK 2003 Global analysis of protein localization in budding yeast. Nature 425:686–691. doi:10.1038/nature02026. 14562095

[34] Christiaens JF, Franco LM, Cools TL, De Meester L, Michiels J, Wenseleers T, Hassan BA, Yaksi E, Verstrepen KJ 2014 The fungal aroma gene *ATF1* promotes dispersal of yeast cells through insect vectors. Cell Rep 9:425–432. doi:10.1016/j.celrep.2014.09.009. 25310977

[35] Rodriguez GM, Tashiro Y, Atsumi S 2014 Expanding ester biosynthesis in *Escherichia coli*. Nat Chem Biol 10:259–265. doi:10.1038/nchembio.1476. 24609358 PMC4411949

[36] Lilly M, Bauer FF, Lambrechts MG, Swiegers JH, Cozzolino D, Pretorius IS 2006 The effect of increased yeast alcohol acetyltransferase and esterase activity on the flavour profiles of wine and distillates. Yeast 23:641–659. doi:10.1002/yea.1382. 16845703

[37] Nancolas B, Bull ID, Stenner R, Dufour V, Curnow P 2017 *Saccharomyces cerevisiae* Atf1p is an alcohol acetyltransferase and a thioesterase in vitro. Yeast 34:239–251. doi:10.1002/yea.3229. 28160314 PMC5484351

[38] Knight MJ, Bull ID, Curnow P 2014 The yeast enzyme Eht1 is an octanoyl-CoA:ethanol acyltransferase that also functions as a thioesterase. Yeast 31:463–474. doi:10.1002/yea.3046. 25308280 PMC4282330

[39] Slagsvold T, Pattni K, Malerød L, Stenmark H 2006 Endosomal and non-endosomal functions of ESCRT proteins. Trends Cell Biol 16:317–326. doi:10.1016/j.tcb.2006.04.004. 16716591

[40] Olmos Y, Carlton JG 2016 The ESCRT machinery: new roles at new holes. Curr Opin Cell Biol 38:1–11. doi:10.1016/j.ceb.2015.12.001. 26775243 PMC5023845

[41] Henne WM, Buchkovich NJ, Emr SD 2011 The ESCRT pathway. Dev Cell 21:77–91. doi:10.1016/j.devcel.2011.05.015. 21763610

[42] Babst M, Katzmann DJ, Snyder WB, Wendland B, Emr SD 2002 Endosome-associated complex, ESCRT-II, recruits transport machinery for protein sorting at the multivesicular body. Dev Cell 3:283–289. doi:10.1016/S1534-5807(02)00219-8. 12194858

[43] Fujiwara D, Kobayashi O, Yoshimoto H, Harashima S, Tamai Y 1999 Molecular mechanism of the multiple regulation of the *Saccharomyces cerevisiae ATF1* gene encoding alcohol acetyltransferase. Yeast 15:1183–1197. doi:10.1002/(SICI)1097-0061(19990915)15:12<1183::AID-YEA444>3.0.CO;2-J. 10487921

[44] O’Connor-Cox ESC, Ingledew WM 1989 Wort nitrogenous sources—their use by brewing yeasts: a review. J Am Soc Brew Chem 47:102–108. doi:10.1094/ASBCJ-47-0102.

[45] Schmidt SA, Dillon S, Kolouchova R, Henschke PA, Chambers PJ 2011 Impacts of variations in elemental nutrient concentration of Chardonnay musts on *Saccharomyces cerevisiae* fermentation kinetics and wine composition. Appl Microbiol Biotechnol 91:365–375. doi:10.1007/s00253-011-3197-3. 21476141

[46] Benatuil L, Perez JM, Belk J, Hsieh CM 2010 An improved yeast transformation method for the generation of very large human antibody libraries. Protein Eng Des Sel 23:155–159. doi:10.1093/protein/gzq002. 20130105

[47] Dawes IW, Hardie ID 1974 Selective killing of vegetative cells in sporulated yeast cultures by exposure to diethyl ether. Mol Gen Genet 131:281–289. doi:10.1007/BF00264859. 4612332

[48] Duitama J, Quintero JC, Cruz DF, Quintero C, Hubmann G, Foulquié-Moreno MR, Verstrepen KJ, Thevelein JM, Tohme J 2014 An integrated framework for discovery and genotyping of genomic variants from high-throughput sequencing experiments. Nucleic Acids Res 42:e44. doi:10.1093/nar/gkt1381. 24413664 PMC3973327

[49] Duitama J, Sánchez-Rodríguez A, Goovaerts A, Pulido-Tamayo S, Hubmann G, Foulquié-Moreno MR, Thevelein JM, Verstrepen KJ, Marchal K 2014 Improved linkage analysis of quantitative trait loci using bulk segregants unveils a novel determinant of high ethanol tolerance in yeast. BMC Genomics 15:207. doi:10.1186/1471-2164-15-207. 24640961 PMC4003806

[50] Edwards MD, Gifford DK 2012 High-resolution genetic mapping with pooled sequencing. BMC Bioinformatics 13(Suppl 6):S8. doi:10.1186/1471-2105-13-S6-S8. PMC3358661 22537047

[51] Ran FA, Hsu PD, Wright J, Agarwala V, Scott DA, Zhang F 2013 Genome engineering using the CRISPR-Cas9 system. Nat Protoc 8:2281–2308. doi:10.1038/nprot.2013.143. 24157548 PMC3969860

[52] DiCarlo JE, Norville JE, Mali P, Rios X, Aach J, Church GM 2013 Genome engineering in *Saccharomyces cerevisiae* using CRISPR-Cas systems. Nucleic Acids Res 41:4336–4343. doi:10.1093/nar/gkt135. 23460208 PMC3627607

[53] Finn RD, Clements J, Arndt W, Miller BL, Wheeler TJ, Schreiber F, Bateman A, Eddy SR 2015 HMMER web server: 2015 update. Nucleic Acids Res 43:W30–W38. doi:10.1093/nar/gkv397. 25943547 PMC4489315

