# Supplementary figures and images for: Polygenic Analysis in Absence of Major Effector ATF1 Unveils Novel Components in Yeast Flavor Ester Biosynthesis
Source: mBio. 2018 Aug 28;9(4):e01279-18. doi: 10.1128/mBio.01279-18 (PMC6113618; doi:10.1128/mBio.01279-18)

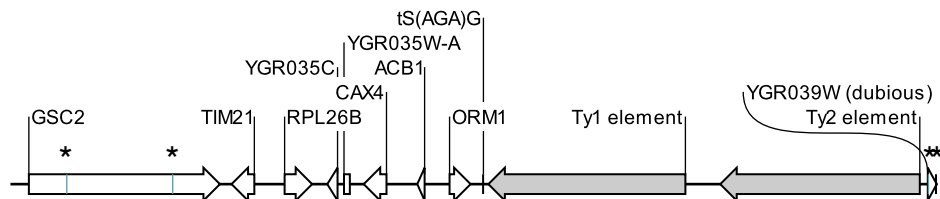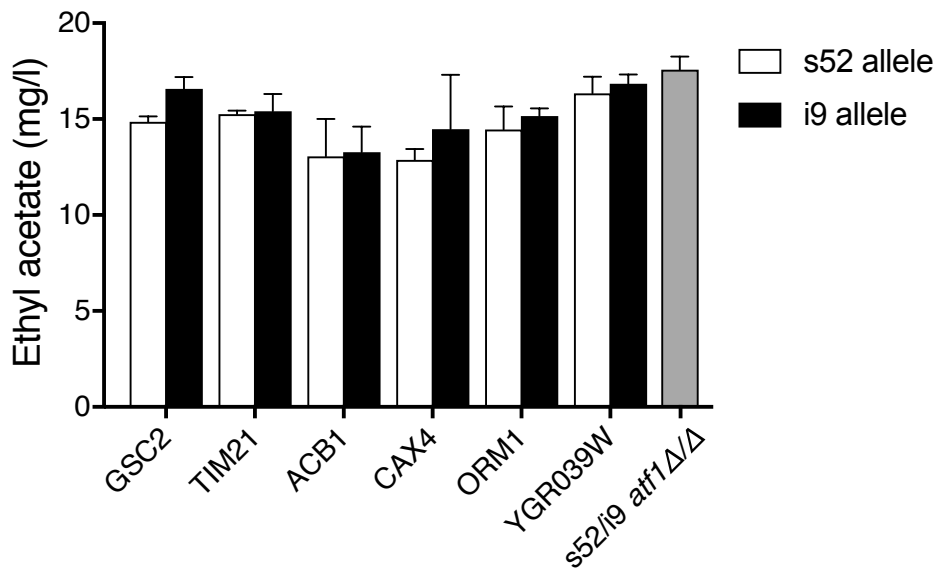

Supplement: FIG S1 [file mbo004184043sf1.pdf]
