# Supplementary material for: Polygenic Analysis in Absence of Major Effector ATF1 Unveils Novel Components in Yeast Flavor Ester Biosynthesis
Source: mBio. 2018 Aug 28;9(4):e01279-18. doi: 10.1128/mBio.01279-18 (PMC6113618; doi:10.1128/mBio.01279-18)
Supplement: TABLE S1 [file mbo004184043st1.docx]

SUPPLEMENTARY INFORMATION

**Supplementary Table 1. Annotated mutations in the QTLs.** All mutations in the QTLs identified are shown with reference to the open reading frames. Linkage intervals (1-LOD drop intervals) for low or high ethyl acetate pools and the position of the blocks investigated with RHA analysis are indicated with grey boxes. The mutations refer to differences between the i9 and s52 strains (i9 -> s52). Genes with structural mutations (nonsense, frame-shift, structural variants, etc.), missense mutations, and mutations in the promoter (Pro.) or terminator (Ter.) regions of genes are indicated. SNVs and structural variants inside the QTLs were checked manually in the assemblies with IGV. Predicted deletions in *MSB2* (in both s52 and i9) and *TPO2* (in i9) based on a subset of reads with abnormal insert sizes were disregarded. Biallelic as well as a small number of multiallelic SNVs in the haploid assemblies of the s52 and i9 strains were disregarded in QTL1 block 1: *MSB2*; QTL2: *YNL019*, *RQC2*, *YNL017C*, *YKL163W*, *YGR130C*, *YGR138C*, and *YGR122C-A*, High-QTL8: *FDH1*, *FEX1*, *YOR390W*, *YOR392W*, *PAU21*, *YOR396C-A*, High-QTL9: *SAP4* (K528 (1575delT)). *RPL17A* contained 3 mutations in the intron located within the gene.

| *atf1Δ* QTLs | |  |  |  |  |  |  |  |
| --- | --- | --- | --- | --- | --- | --- | --- | --- |
| **QTL1** |  |  |  |  |  |  |  |  |
| **Locus** | **ORF** | **Nonsense, frameshift, structural variants, etc.** | **Missense** | **Pro.** | **Ter.** | **Low EA linkage** | **High EA linkage** | **Bulk deletion (RHA)** |
| YGR006W | PRP18 |  |  | X |  |  | chrVII: |  |
| YGR007W | ECT1 |  | Q40K, T57S | X | X |  | 504.6-580.4 |  |
| YGR008C | STF2 |  |  | X |  |  |  |  |
| YGR010W | NMA2 |  |  | X |  | chrVII: |  | Block 1 |
| YGR012W | MCY1 |  | R32K, I49V | X |  | 509.7-591.0 |  | chrVII: |
| YGR013W | SNU71 |  | K61N, M396I | X |  |  |  | 511.167- |
| YGR014W | MSB2 |  | F141L, V152L, 202_203insS, F323L, A380T, N381D, D455G, T503S, A520V, N745S, N779S, A970T, V1021L, T1038A, L1067V | X | X |  |  | 531.016 |
| YGR015C |  | K179*fs*(532delT) -> protein truncation in s52 | L4V, I14R, Q154R |  |  |  |  |  |
| YGR017W |  |  | N158E | X |  |  |  |  |
| YGR019W | UGA1 |  |  | X |  |  |  |  |
| YGR021W |  |  |  | X |  |  |  |  |
| YGR023W | MTL1 |  | T158I, T294I, S732F | X | X |  |  |  |
| YGR025W |  |  |  |  |  |  |  | Block 2 |
| YGR024C | THG1 |  |  |  |  |  |  | chrVII: |
| YGR026W |  |  |  |  |  |  |  | 531.327-547.820 |
| YGR027C | RPS25A |  |  |  |  |  |  |  |
| YGR027W-A and YGR027W-B |  | Rearrangement/deletion of Ty elements (6177 and 6139 bps in s52 and i9, respectively) |  |  |  |  |  |  |
| YGR028W | MSP1 |  |  | X |  |  |  |  |
| YGR029W | ERV1 |  | G57D | X | X |  |  |  |
| YGR031W | IMO32 |  | M2I, E65V, S244F | X |  |  |  |  |
| YGR030C | POP6 |  | S11N, I68M, D101G, S105N |  |  |  |  |  |
| YGR031C-A | NAG1 |  | D44N |  |  |  |  |  |
| YGR032W | GSC2 |  | C382R, V1424A | X |  |  |  | Block 3 |
| YGR034W | RPL26B |  |  | X |  |  |  | chrVII: |
| YGR036C | CAX4 |  |  | X |  |  |  | 547.217-574.317 |
| YGR038W | ORM1 |  |  | X | X |  |  |  |
| YGR039W |  |  | E15G, L89F | X |  |  |  |  |
| YGR038-A and YGR038-B |  | Deletion of Ty1 element (3999 and 5700 bps in s52 and i9, respectively) |  |  |  |  |  |  |
| YGR040W | KSS1 |  |  | X |  |  |  | Block 4 |
| YGR041W | BUD9 |  | K16T, A27T, N132S, S260N, R298K, S321N, T345N, S406G, I480V | X |  |  |  | chrVII: |
| YGR042W | MTE1 |  | N85S | X |  |  |  | 575.179-593.325 |
| YGR043C | NQM1 |  |  | X |  |  |  |  |
| YGR044C | RME1 |  | S278A |  | X |  |  |  |
| YGR046W | TAM41 |  | S383R |  |  |  |  |  |
|  |  |  |  |  |  |  |  |  |
| **QTL2** |  |  |  |  |  |  |  |  |
| **Locus** | **ORF** | **Nonsense, frameshift, structural variants, etc.** | **Missense** | **Pro.** | **Ter.** | **Low EA linkage** | **High EA linkage** | **Bulk deletion (RHA)** |
| YPL036W | PMA2 |  | I92V, A764T, A838V | X | X | chrXVI: |  |  |
| YPL034W |  |  |  | X |  | 484.2-570.0 |  |  |
| YPL035C | (dubious ORF) |  | S106M | X |  |  |  |  |
| YPL033C | SRL4 |  | A69V, K124Q, R235H | X |  |  |  |  |
| YPL032C | SVL3 |  | 418-419delAQ, S608P | X |  |  |  |  |
| YPL030W | TRM44 |  | D19G, M122I, S223F | X |  |  |  |  |
| YPL029W | SUV3 |  | D176N, K410R | X |  |  |  |  |
| YPL028W | ERG10 |  |  | X |  |  |  |  |
| YPL027W | SMA1 |  | N27K, N31S, S47N, V115I, L194F |  | X |  |  |  |
| YPL026C | SKS1 |  | S92L, L310S, E399G, N403K, A407E, N486K, D501N | X |  |  |  |  |
| YPL025C | (dubious ORF) | M63*fs*(187delA) -> protein truncation in s52 |  |  |  |  |  |  |
| YPL024W | RMI1 |  |  | X | X |  |  |  |
| YPL023C | MET12 |  | S388F |  |  |  |  |  |
| YPL022W | RAD1 |  | P37L, D175G, M407I, V988I, L1046F | X | X |  |  |  |
| YPL021W | ECM23 |  | P93L, S175T, G176E | X | X |  |  |  |
| YPL020C | ULP1 |  | H106R, S199N, K246E | X | X |  |  |  |
| YPL019C | VTC3 |  | N84H, V244I, D394E, N608S, V612I, S746F, G808E |  |  |  |  |  |
| YPL018W | CTF19 |  | D22N | X |  |  |  |  |
| YPL017C | IRC15 |  | D3G, I14F, V39A, I164V, L254V, V267I, K399R |  |  |  |  |  |
| YPL016W | SWI1 |  | G242S, S310A, E744K, K1314E | X | X |  |  |  |
| YPL015C | HST2 |  | V168A, K216Q, R296G, D337G |  | X |  |  |  |
| YPL014W | CIP1 |  | N54D | X |  |  |  | Block 1 |
| YPL012W | RRP12 |  | E688K | X | X |  |  | chrVII: |
| YPL009C | RQC2 | K1037*fs* (3102_3103insT) -> protein extension in s52 | N294I, R627K |  |  |  | chrXVI: 536.6-569.4 | 527.628-548.112 |
| YPL008W | CHL1 |  |  | X |  |  |  |  |
| YPL007C | TCF8 |  | E141K, A179T, V209L |  |  |  |  |  |
| YPL006W | NCR1 |  |  | X |  |  |  |  |
| YPL005W | AEP3 |  | F244L, R472Q | X |  |  |  | Block 2 |
| YPL003W | ULA1 |  | I189T | X |  |  |  | chrVII: |
| YPL002C | SNF8 | E148Stop |  |  |  |  |  | 548.539-554.307 |
| YPR001W | CIT3 |  | L134F |  |  |  |  | Block 3 |
| YPR002W | PDH1 |  |  | X | X |  |  | chrVII: |
| YPR002C-A | (dubious ORF) |  |  | X | X |  |  | 556.401-569.031 |
| YPR003C |  |  |  | X |  |  |  |  |
| YPR004C | AIM45 | K342*fs* (1022delT) -> protein extension in s52 |  | X |  |  |  |  |
| YPR005C | HAL1 | V90*fs* (270delC) -> protein truncation in s52 |  | X |  |  |  |  |
| YPR006C | ICL2 |  | S322Y, K329Q, M406I |  |  |  |  |  |
|  |  |  |  |  |  |  |  |  |
| **Low-QTL3** | |  |  |  |  |  |  |  |
| **Locus** | **ORF** | **Nonsense, frameshift, structural variants, etc.** | **Missense** | **Pro.** | **Ter.** | **Low EA linkage** | **High EA linkage** |  |
| YNL035C |  |  | D235E, D264N, N284I |  | X | chrXIV: |  |  |
| YNL034W |  | Partial gene deletion in s52 (1900bps) |  |  |  | 565.2-613.6 |  |  |
| YNL033W |  |  | S222P, A262V | X |  |  |  |  |
| YNL032W | SIW14 |  |  | X | X |  |  |  |
| YNL030W | HHF2 |  |  | X | X |  |  |  |
| YNL029C | KTR5 |  | A49T, G286D, V317G, V502A |  |  |  |  |  |
| YNL027W | CRZ1 |  | N37T |  |  |  |  |  |
| YNL026W | SAM50 |  | N2T, M65V, P228Q | X | X |  |  |  |
| YNL025C | SSN8 |  | H13Q | X | X |  |  |  |
| YNL024C-A | KSH1 |  |  | X | X |  |  |  |
| YNL024C | EFM6 |  |  | X |  |  |  |  |
| YNL023C | FAP1 |  | T35A, G110R, N198K, I423T, R602K, T659R, L753S, V768I | X |  |  |  |  |
| YNL022C | RCM1 |  | D357G |  |  |  |  |  |
| YNL021W | HDA1 |  | I416M, T545K | X | X |  |  |  |
| YNL020C | ARK1 |  | V97M, N391D, L522S, I593T | X |  |  |  |  |
| YNL019C |  |  | S222P | X |  |  |  |  |
| YNL018C |  | Partially absent in i9 |  | X |  |  |  |  |
| YNL016W | PUB1 |  | Q323R | X |  |  |  |  |
| YNL017C | (dubious ORF) | Deletion (with poor assembly) |  |  |  |  |  |  |
| YNL015W | PBI2 | Downstream deletion in s52 (20bps at -123_-143) |  | X |  |  |  |  |
| YNL014W | HEF3 |  |  | X |  |  |  |  |
| YNL012W | SPO1 |  | D174N, G185E, P210T, T248K, G585E | X | X |  |  |  |
|  |  |  |  |  |  |  |  |  |
| **Low-QTL4** | |  |  |  |  |  |  |  |
| **Locus** | **ORF** | **Nonsense, frameshift, structural variants, etc.** | **Missense** | **Pro.** | **Ter.** | **Low EA linkage** | **High EA linkage** |  |
| YKL182W | FAS1 |  | S645N, D1216E, A1504V, A1715V, V1970Y |  |  | chrXI: 102.5-163.9 |  |  |
| YKL181W | PRS1 |  |  | X |  |  |  |  |
| YKL180W | RPL17A |  |  | X | X |  |  |  |
| YKL179C | COY1 |  | I318V, I423T, L489I, G557E | X |  |  |  |  |
| YKL178C | STE3 |  | M408T | X |  |  |  |  |
| YKL176C | LST4 |  | T700S, S784N |  |  |  |  |  |
| YKL175W | ZRT3 |  | D149G, T158S | X |  |  |  |  |
| YKL174C | TPO5 |  | S29T, G590E |  |  |  |  |  |
| YKL173W | SNU114 |  | L439I, I652M, K725R, T802A | X |  |  |  |  |
| YKL171W | NNK1 |  | Q9R, N107D, S226G, R575T | X |  |  |  |  |
| YKL170W | MRPL38 |  |  | X |  |  |  |  |
| YKL168C | KKQ8 |  | S341P, N347S, E352G, Q534R | X |  |  |  |  |
| YKL166C | TPK3 |  | T208I, G375R |  |  |  |  |  |
| YKL165C-A | (dubious ORF) |  | N29S, I40T | X |  |  |  |  |
| YKL165C | MCD4 |  | T387I, I440T | X |  |  |  |  |
| YKL164C | PIR1 |  | G109S, D284A | X |  |  |  |  |
| YKL163W | PIR3 |  | V271I | X | X |  |  |  |
| YKL162C-A | (dubious ORF) |  |  | X | X |  |  |  |
| YKL162C |  |  | E228K, Y390D | X | X |  |  |  |
| YKL161C | KDX1 |  | F146S, S369G | X |  |  |  |  |
| YKL160W | ELF1 |  |  | X |  |  |  |  |
| YKL157W | AEP2 |  | R65S, T149I, E167G, S246A, S453G | X |  |  |  |  |
| YKL159C | RCN1 |  |  | X |  |  |  |  |
| YKL156C-A |  |  |  | X |  |  |  |  |
| YKL154W | SRP102 |  |  | X | X |  |  |  |
| YKL153W |  |  |  | X |  |  |  |  |
|  |  |  |  |  |  |  |  |  |
| **High-QTL5** | |  |  |  |  |  |  |  |
| **Locus** | **ORF** | **Nonsense, frameshift, structural variants, etc.** | **Missense** | **Pro.** | **Ter.** | **Low EA linkage** | **High EA linkage** |  |
| YAL033W | POP5 |  |  | X | X |  | chrI: |  |
| YAL032C | PRP45 | Stop380Q -> protein extension in s52 | Q13E, N103S, D191G, I288V |  |  |  | 82.1-129.3 |  |
| YAL031W-A | (dubious ORF) | F11*fs* (33_34 insT ) -> protein truncation in s52 |  |  |  |  |  |  |
| YAL031C | GIP4 |  | E338D, Q424R, S481T, H589Q, K647N, W725L | X |  |  |  |  |
| YAL029C | MYO4 |  | F52L, A536T, E736A, V856I, S864R, V904I, A926V, G1002V, T1071A, S1176G, N1366K, S1454G, K1471T |  |  |  |  |  |
| YAL028W | FRT2 |  | G12D, D19S, A222T, I235V, A286V | X |  |  |  |  |
| YAL027W | SAW1 |  | I27M, I77V, I111T, P214L | X |  |  |  |  |
| YAL026C-A | (dubious ORF) | Stop146Q -> protein extention in s52 | T91S, P122L, T138SR, T145A |  |  |  |  |  |
| YAL026C | DRS2 |  | S30T, L427M | X |  |  |  |  |
| YAL025C | MAK16 |  |  | X |  |  |  |  |
| YAL024C | LTE1 | L121*fs* (357delA ) -> protein truncation in s52 | V25L | X |  |  |  |  |
| YAL023C | PMT2 |  |  | X |  |  |  |  |
| YAL022C | FUN26 |  | M182V | X |  |  |  |  |
| YAL021C | CCR4 |  | N727S, K835R |  |  |  |  |  |
| YAL019W-A | (dubious ORF) |  | C24R, R114G | X |  |  |  |  |
| YAL020C | ATS1 |  | E130G, V172I, T276I |  |  |  |  |  |
| YAL019W | FUN30 |  | S4P, E224D | X |  |  |  |  |
| YAL018C | LDS1 |  | S152C |  |  |  |  |  |
| YAL017W | PSK1 |  | V230S |  |  |  |  |  |
| YAL016W | TPD3 |  |  | X | X |  |  |  |
| YAL015C | NTG1 |  |  | X |  |  |  |  |
|  |  |  |  |  |  |  |  |  |
| **High-QTL6** | |  |  |  |  |  |  |  |
| **Locus** | **ORF** | **Nonsense, frameshift, structural variants, etc.** | **Missense** | **Pro.** | **Ter.** | **Low EA linkage** | **High EA linkage** |  |
| YGR232W | NAS6 |  | 3_4insN, G134R, P166A | X | X |  | chrVII: |  |
| YGR233C | PHO81 | K238*fs*(714insA) -> full length protein in s52 | M45L | X |  |  | 952.6-999.0 |  |
| YGR234W | YHB1 |  | E153D | X |  |  |  |  |
| YGR236C | SPG1 |  |  | X |  |  |  |  |
| YGR237C |  |  |  | X |  |  |  |  |
| YGR238C | KEL2 |  |  | X |  |  |  |  |
| YGR239C | PEX21 |  | P225S | X |  |  |  |  |
| YGR240C | PFK1 |  | K891E |  |  |  |  |  |
| YGR240C-A |  |  | T47I, M65I |  |  |  |  |  |
| YGR241C | YAP1802 |  | N295I, R317K, E348D, M510V, L518H |  |  |  |  |  |
| YGR243W | MPC3 |  | I114M | X | X |  |  |  |
| YGR244C | LSC2 |  |  | X |  |  |  |  |
| YGR245C | SDA1 |  |  | X |  |  |  |  |
| YGR246C | BRF1 |  | S529N |  |  |  |  |  |
| YGR247W | CPD1 |  |  | X |  |  |  |  |
| YGR248W | SOL4 |  | A125T, I175V | X | X |  |  |  |
| YGR249W | MGA1 | F32*fs* (105delT) -> full length protein in s52 | P3R | X | X |  |  |  |
|  |  |  |  |  |  |  |  |  |
| **High-QTL7** | |  |  |  |  |  |  |  |
| **Locus** | **ORF** | **Nonsense, frameshift, structural variants, etc.** | **Missense** | **Pro.** | **Ter.** | **Low EA linkage** | **High EA linkage** |  |
| YJL073W | JEM1 |  | S546P |  |  |  | chrX: |  |
| YJL071W | ARG2 |  | K554R |  |  |  | 301.8-345.3 |  |
| YJL070C |  |  | V40A, P61S, 213delY, S427N | X |  |  |  |  |
| YJL069C | UTP18 |  | E75D, T102A, F313L | X |  |  |  |  |
| YJL068C |  |  | L252V |  |  |  |  |  |
| YJL066C | MPM1 |  | N247S |  |  |  |  |  |
| YJL064W |  |  |  | X |  |  |  |  |
| YJL065C | DLS1 |  | T135S | X |  |  |  |  |
| YJL063C | MRPL8 |  | A64V |  |  |  |  |  |
| YJL062W | LAS21 |  |  | X |  |  |  |  |
| YJL061W | NUP82 |  |  | X |  |  |  |  |
| YJL060W | BNA3 |  |  | X |  |  |  |  |
| YJL059W |  |  | K223R, E262G |  |  |  |  |  |
| YJL057C | IKS1 |  | 266delE, T440A | X |  |  |  |  |
| YJL056C | ZAP1 |  | T22I, P186S, Q403H, I675T, R676H |  |  |  |  |  |
| YJL054W | TIM54 |  | T295M | X |  |  |  |  |
| YJL053W | PEP8 |  | 214_215insASDN, G229A | X | X |  |  |  |
| YJL052W | TDH1 |  |  | X |  |  |  |  |
| YJL051W | IRC8 |  | A227S, H232Y, V440E, T485N, G491D, G616S | X |  |  |  |  |
| YJL050W | MTR4 |  | T69M, T702I, D847E, H884R | X |  |  |  |  |
|  |  |  |  |  |  |  |  |  |
| WT QTLs | |  |  |  |  |  |  |  |
| **Low-QTL8** | |  |  |  |  |  |  |  |
| **Locus** | **ORF** | **Nonsense, frameshift, structural variants, etc.** | **Missense** | **Pro.** | **Ter.** | **Low EA linkage** | **High EA linkage** |  |
| YOL066C | RIB2 |  |  | X |  | chrXV: |  |  |
| YOL065C | INP54 |  | L55V, K115N | X |  | 204.6-224.8 |  |  |
| YOL064C | MET22 |  | S40N, S63N, N105D |  |  |  |  |  |
| YOL063C | CRT10 | Q957Stop -> Truncation of 1 aa glutamine (Q) in s52 | M50I, V51I, S100F, Q144P, V190I, A370V, H408D, S429L, F605S, L653I, P729A, V755A, A876P, E953G |  |  |  |  |  |
| YOL062C | APM4 |  | S4G, T163A |  |  |  |  |  |
| YOL061W | PRS5 |  |  | X | X |  |  |  |
| YOL060C | MAM3 |  | I375M, I556V, E633D, S661N |  |  |  |  |  |
| YOL059W | GPD2 |  | Q58H, G376E | X |  |  |  |  |
| YOL058W | ARG1 |  |  | X |  |  |  |  |
| YOL057W |  |  | R45K, N70D, K116E, M612I | X |  |  |  |  |
| YOL056W | GPM3 |  | G251D | X |  |  |  |  |
|  |  |  |  |  |  |  |  |  |
| **Low-QTL9** | |  |  |  |  |  |  |  |
| **Locus** | **ORF** | **Nonsense, frameshift, structural variants, etc.** | **Missense** | **Pro.** | **Ter.** | **Low EA linkage** | **High EA linkage** |  |
| YGR122W |  |  |  |  | X | chrVII: |  |  |
| YGR122C-A | (Dubious ORF) |  |  | X | X | 733.8-772.3 |  |  |
| YGR123C | PPT1 |  |  | X |  |  |  |  |
| YGR124W | ASN2 |  | A500V | X |  |  |  |  |
| YGR125W |  |  | L981I | X |  |  |  |  |
| YGR127W |  |  |  |  | X |  |  |  |
| YGR128C | UTP8 |  | T707A |  |  |  |  |  |
| YGR130C |  |  | I572M |  |  |  |  |  |
| YGR131W | FHN1 |  |  | X | X |  |  |  |
| YGR133W | PEX4 |  | Q132H |  |  |  |  |  |
| YGR134W | CAF130 |  | S274G, K423R | X |  |  |  |  |
| YGR137W |  |  |  |  | X |  |  |  |
| YGR138C | TPO2 |  |  | X |  |  |  |  |
| YGR139W | (Dubious ORF) | E16*fs* (48delA) -> truncated protein in s52 |  |  |  |  |  |  |
| YGR140W | CBF2 |  | K10N | X |  |  |  |  |
| YGR142W | BTN2 |  |  | X |  |  |  |  |
|  |  |  |  |  |  |  |  |  |
| **High-QTL10** | |  |  |  |  |  |  |  |
| **Locus** | **ORF** | **Nonsense, frameshift, structural variants, etc.** | **Missense** | **Pro.** | **Ter.** | **Low EA linkage** | **High EA linkage** |  |
| YJL225W-A |  | Poor coverage/assembly |  |  |  |  | chrX: |  |
| YJL225C |  | Poor coverage/assembly |  |  |  |  | (0)-9.8 |  |
| YJL223C | PAU1 | Poor coverage/assembly |  |  |  |  |  |  |
| YJL222W-A |  | Completely absent in s52 |  |  |  |  |  |  |
| YJL222W | VTH2 | Completely absent in s52 |  |  |  |  | Downstream: 11.475-16.124 |  |
|  |  |  |  |  |  |  |  |  |
| **High-QTL11** | |  |  |  |  |  |  |  |
| **Locus** | **ORF** | **Nonsense, frameshift, structural variants, etc.** | **Missense** | **Pro.** | **Ter.** | **Low EA linkage** | **High EA linkage** |  |
| YNR034W | SOL1 |  |  | X | X |  | chrXIV: |  |
| YNR034W-A | EGO4 |  |  | X | X |  | 689.9-694.5 |  |
| YNR035C | ARC35 |  | S116L | X |  |  |  |  |
|  |  |  |  |  |  |  |  |  |
| **High-QTL12** | |  |  |  |  |  |  |  |
| **Locus** | **ORF** | **Nonsense, frameshift, structural variants, etc.** | **Missense** | **Pro.** | **Ter.** | **Low EA linkage** | **High EA linkage** |  |
| YOR389W |  | Poor coverage/assembly |  |  |  |  | chrXV: |  |
| YOR390W | FEX1 |  |  | X | X |  | 1,072.2- |  |
| YOR392W | (Dubious ORF) |  |  | X |  |  | (1,087.0) |  |
| YOR391C | HSP33 | Poor coverage in s52 |  |  |  |  |  |  |
| YOR393W | ERR1 |  |  | X |  |  |  |  |
| YOR394W | PAU21 |  | V28A | X | X |  |  |  |
| YOR394C-A |  | Poor coverage in i9 |  |  |  |  |  |  |
| YOR396W | YRF1-8 | Poor coverage | V425M | X |  |  |  |  |
| YOR396C-A |  |  |  | X |  |  |  |  |
|  |  |  |  |  |  |  |  |  |
| **High-QTL13** | |  |  |  |  |  |  |  |
| **Locus** | **ORF** | **Nonsense, frameshift, structural variants, etc.** | **Missense** | **Pro.** | **Ter.** | **Low EA linkage** | **High EA linkage** |  |
| YGL229C | SAP4 |  | E47G, A83G, E388K, 411delH, K486N, 549delD, F586L, P587S, S647N |  |  |  | chrVII: 65.0-95.5 |  |
| YGL228W | SHE10 |  | T9I, S28N, L138I, F146S, T179I, R205H, G263D, E317K | X |  |  |  |  |
| YGL227W | VID30 |  | Y37H, K77Q, V237I, S443F, V882I | X |  |  |  |  |
| YGL226W | MTC3 |  |  | X |  |  |  |  |
| YGL226C-A | OST5 |  |  | X |  |  |  |  |
| YGL225W | VGR4 |  |  | X | X |  |  |  |
| YGL224C | SDT1 |  | V98I, P277S | X |  |  |  |  |
| YGL223C | COG1 |  | Y235H, Q288K, T388M | X |  |  |  |  |
| YGL222C | EDC1 |  | N95K |  |  |  |  |  |
| YGL220W | BOL2 |  | G26R |  |  |  |  |  |
| YGL216W | KIP3 |  |  | X |  |  |  |  |
| YGL215W | CLG1 |  | L84P | X |  |  |  |  |
| YGL214W |  |  |  | X |  |  |  |  |
| YGL213C | SKI8 |  | A356G |  |  |  |  |  |
| YGL212W | VAM7 | G7*fs* (21_22insG) -> protein truncation in s52 |  |  |  |  |  |  |
| YGL211W | BOL2 |  |  | X |  |  |  |  |
| YGL210W | YPT32 |  |  |  | X |  |  |  |
|  |  |  |  |  |  |  |  |  |
| **High-QTL14** | |  |  |  |  |  |  |  |
| **Locus** | **ORF** | **Nonsense, frameshift, structural variants, etc.** | **Missense** | **Pro.** | **Ter.** | **Low EA linkage** | **High EA linkage** |  |
| YJR147W | HMS2 |  | L39F, G77D, K152R, L157V, D209N, P312S | X |  |  | chrX: 703.5- |  |
| YJR148W | BAT2 |  | S169C | X |  |  | (745.751) |  |
| YJR149W |  |  | M130V, S357N | X |  |  |  |  |
| YJR150C | DAN1 |  | I272V | X |  |  |  |  |
| YJR151C | DAN4 | Deletion of 133 aa in i9 (381V_514Tdel) |  |  |  |  |  |  |
| YJR151W-A |  |  |  | X |  |  |  |  |
| YJR152W | DAL5 |  | A33V, V174I | X | X |  |  |  |
| YJR153W | PGU1 |  | G293S | X | X |  |  |  |
| YJR154W |  |  |  | X |  |  |  |  |
| YJR155W | AAD10 | Partially absent s52 |  |  |  |  |  |  |
| YJR157W |  | Completely absent in s52 |  |  |  |  |  |  |
| YJR158W | HXT16 | Completely absent in s52 |  |  |  |  |  |  |
| YJR159W | SOR1 | Completely absent in s52 |  |  |  |  |  |  |
| YJR160C | MPH3 | Completely absent in s52 |  |  |  |  |  |  |
| YJR161C | COS5 | Poor coverage/assembly in s52 |  |  |  |  |  |  |
| YJR162C |  | ORF in telomere region |  |  |  |  |  |  |
