# Supplementary material for: Polygenic Analysis in Absence of Major Effector ATF1 Unveils Novel Components in Yeast Flavor Ester Biosynthesis
Source: mBio. 2018 Aug 28;9(4):e01279-18. doi: 10.1128/mBio.01279-18 (PMC6113618; doi:10.1128/mBio.01279-18)
Supplement: TABLE S2 [file mbo004184043st2.docx]

SUPPLEMENTARY INFORMATION

**Supplementary Table 2. Aroma compounds produced in fermentations by strains used to confirm the causative frame shift and nonsense mutations in *EAT1* and *SNF8*.** Aroma profiles produced in fermentations by the **A.** i9 *atf1Δ*, **B.** s52 *atf1Δ*, **C.** i9 and **D.** s52 strains engineered with *EAT1* frame-shift and *SNF8* nonsense mutations. The significance of any deviation from the mean of the parent or the engineered strains is indicated as follows: ns p>0.05, *p≤0.05, **p≤0.01, ***p≤0.001. bd = below detection. All fermentations were carried out with four replicates and aroma production values are shown +/- s.d.

| **A** |  |  |  |  |
| --- | --- | --- | --- | --- |
|  | i9 *atf1*Δ | *eat1*^K179fs^ | *snf8^E148^* | *eat1^K179fs^ snf8^E148^* |
| Acetaldehyde | 2.76±0.24 | 3.61±1.17 (ns) | 5.63±0.71 (**) | 7.39±3.53 (ns) |
| Ethyl acetate | 10.5±0.5 | 7.3±0.3 (***) | 7.2±0.2 (***) | 4.4±0.2 (***) |
| Isoamyl acetate | 0.15±0.01 | 0.15±0.03 (ns) | 0.15±0.01 (ns) | 0.14±0.02 (ns) |
| Ethyl hexanoate | 0.23±0.04 | 0.22±0.03 (ns) | 0.28±0.05 (ns) | 0.19±0.02 (ns) |
| Ethyl octanoate | 0.46±0.04 | 0.44±0.03 (ns) | 0.35±0.01 (**) | 0.30±0.02 (**) |
| Ethyl decanoate | 0.29±0.09 | 0.33±0.01 (ns) | 0.24±0.02 (ns) | 0.24±0.03 (ns) |
| Isobutanol | 15.7±1.0 | 18.6±1.2 (ns) | 12.7±0.5 (**) | 15.1±0.7 (ns) |
| Isoamyl alcohol | 72.4±2.8 | 78.3±6.8 (ns) | 61.2±2.7 (**) | 65.9±4.0 (ns) |
|  |  |  |  |  |
| **B** |  |  |  |  |
|  | s52 *atf1*Δ | *EAT1^fs^*^179K^ | *SNF8*^*148E^ | *EAT1*^fs179K^ *SNF8*^*148E^ |
| Acetaldehyde | 10.81±2.97 | 9.42±1.96 (ns) | 4.92±1.36 (ns) | 6.25±1.67 (ns) |
| Ethyl acetate | 2.4±0.2 | 4.5±0.1 (***) | 3.5±0.1 (***) | 5.6±0.2 (***) |
| Isoamyl acetate | 0.12±0.01 | 0.12±0.01 (ns) | 0.23±0.03 (**) | 0.23±0.01 (***) |
| Ethyl hexanoate | 0.14±0.02 | 0.15±0.02 (ns) | 0.15±0.03 (ns) | 0.12±0.03 (ns) |
| Ethyl octanoate | 0.21±0.03 | 0.22±0.01 (ns) | 0.24±0.03 (ns) | 0.18±0.02 (ns) |
| Ethyl decanoate | 0.18±0.01 | 0.21±0.01 (*) | 0.22±0.04 (ns) | 0.15±0.04 (ns) |
| Isobutanol | 65.9±3.7 | 54.5±1.5 (**) | 108.6±5.5 (***) | 85.4±2.5 (***) |
| Isoamyl alcohol | 174.5±7.3 | 140.9±4.0 (**) | 333.7±13.2 (***) | 307.3±6.8 (***) |
|  |  |  |  |  |

| **C** |  |  |  |  |
| --- | --- | --- | --- | --- |
|  | i9 | *eat1*^K179fs^ | *snf8^E148*^* | *eat1^K179fs^ snf8^E148*^* |
| Acetaldehyde | 7.72±1.50 | 12.11±0.78 (*) | 13.67±0.80 (**) | 16.23±0.81 (***) |
| Ethyl acetate | 47.3±3.3 | 55.3±2.2 (*) | 30.2±0.8 (***) | 39.0±0.9 (*) |
| Isobutyl acetate | 0.2±0.0 | 0.4±0.0 (**) | 0.2±0.0 (*) | 0.3±0.0 (ns) |
| Isoamyl acetate | 2.5±0.5 | 3.6±0.3 (*) | 2.4±0.1 (ns) | 3.0±0.2 (ns) |
| Ethyl hexanoate | 0.18±0.05 | 0.24±0.04 (ns) | 0.18±0.02 (ns) | 0.15±0.02 (ns) |
| Ethyl octanoate | 0.24±0.02 | 0.22±0.01 (ns) | 0.29±0.05 (ns) | 0.28±0.02 (ns) |
| Ethyl decanoate | 0.29±0.01 | 0.29±0.01 (ns) | 0.20±0.00 (***) | 0.18±0.01 (***) |
| Isobutanol | 16.97±2.30 | 25.38±2.98 (*) | 14.76±1.65 (ns) | 19.70±0.17 (ns) |
| Isoamyl alcohol | 47.48±2.81 | 59.98±2.00 (**) | 52.55±2.88 (ns) | 59.95±2.56 (**) |
| IAAc/Alc ratio | 0.053±0.012 | 0.060±0.005 (ns) | 0.045±0.004 (ns) | 0.051±0.002 (ns) |
|  |  |  |  |  |
| **D** |  |  |  |  |
|  | i9 | *eat1*^K179fs^ | *snf8^E148*^* | *eat1^K179fs^ snf8^E148*^* |
| Acetaldehyde | 9.09±1.29 | 7.96±0.70 (ns) | 7.01±2.57 (ns) | 8.30±2.16 (ns) |
| Ethyl acetate | 15.8±0.9 | 19.9±1.5 (*) | 15.5±0.9 (ns) | 16.9±1.3 (ns) |
| Isobutyl acetate | 0.4±0.0 | 0.4±0.1 (ns) | 0.4±0.0 (ns) | 0.4±0.0 (ns) |
| Isoamyl acetate | 3.3±0.3 | 3.7±0.8 (ns) | 4.2±0.5 (ns) | 4.5±0.4 (*) |
| Ethyl hexanoate | 0.10±0.01 | 0.12±0.01 (ns) | 0.09±0.01 (ns) | 0.10±0.01 (ns) |
| Ethyl octanoate | 0.27±0.01 | 0.35±0.03 (*) | 0.19±0.03 (*) | 0.16±0.03 (**) |
| Ethyl decanoate | 0.16±0.00 | 0.20±0.02 (ns) | 0.17±0.04 (ns) | 0.13±0.03 (ns) |
| Isobutanol | 78.37±3.99 | 58.19±1.87 (**) | 151.00±13.40 (***) | 141.61±9.05 (***) |
| Isoamyl alcohol | 168.00±3.49 | 134.40±14.69 (*) | 315.49±7.32 (***) | 297.86±7.79 (***) |
| IAAc/Alc ratio | 0.020±0.002 | 0.027±0.004 (ns) | 0.013±0.002 (*) | 0.015±0.002 (ns) |
