# Supplementary material for: Polygenic Analysis in Absence of Major Effector ATF1 Unveils Novel Components in Yeast Flavor Ester Biosynthesis
Source: mBio. 2018 Aug 28;9(4):e01279-18. doi: 10.1128/mBio.01279-18 (PMC6113618; doi:10.1128/mBio.01279-18)
Supplement: TABLE S3 [file mbo004184043st3.docx]

SUPPLEMENTARY INFORMATION

**Supplementary Table 3. Aroma compounds produced by strains with overexpression of *EAT1* and *IMO32* from the multicopy p426 plasmid and the constitutive *TEF1* promoter.** The *EAT1* and *IMO32* genes were PCR amplified from the s52 or i9 strains and cloned into the multicopy p426 plasmid under a constitutive TEF1 promoter. The production of aroma metabolites was analysed by GC-FID in the hybrid strains **A.** without *ATF1* (s52/i9 *atf1Δ*/*Δ*) or **B.** with *ATF1* (s52/i9) transformed with overexpression plasmids. Fermentations were carried out in YP250-10%glucose with 200 mg/L hygromycin to maintain the plasmids. The table shows average aroma production values from four fermentation replicates ± s.d, and the significance of any deviation from the mean of the strains containing empty plasmid.

| **A** |  |  |  |  |  |
| --- | --- | --- | --- | --- | --- |
|  | Empty plasmid | *EAT1*^s52^ | *EAT1*^i9^ | *IMO32*^s52^ | *IMO32*^i9^ |
| Acetaldehyde | 20.1±19.9 | 3.6±3.7 (ns) | 3.5±1.9 (ns) | 3.9±4.4 (ns) | 31.2±20.2 (ns) |
| Ethyl acetate | 13.1±0.9 | 40.8±1.7 (***) | 97.8±3.5 (***) | 11.1±0.4 (ns) | 10.9±0.5 (*) |
| Isoamyl acetate | 0.21±0.06 | 0.34±0.16 (ns) | 0.36±0.03 (*) | 0.16±0.02 (ns) | 0.15±0.01 (ns) |
| Isobutyl acetate | 0.03±0.00 | 0.05±0.01 (**) | 0.14±0.01 (***) | 0.02±0.00 (*) | 0.03±0.00 (ns) |
| Ethyl hexanoate | 0.28±0.03 | 0.31±0.04 (ns) | 0.34±0.04 (ns) | 0.33±0.03 (ns) | 0.36±0.05 (ns) |
| Ethyl octanoate | 0.19±0.01 | 0.18±0.01 (ns) | 0.21±0.01 (ns) | 0.22±0.02 (ns) | 0.15±0.02 (ns) |
| Ethyl decanoate | 0.13±0.02 | 0.13±0.01 (ns) | 0.15±0.02 (ns) | 0.14±0.01 (ns) | 0.08±0.02 (ns) |
| Isoamyl alcohol | 113.3±7.5 | 106.2±4.2 (ns) | 131.4±4.3 (*) | 113.6±1.5 (ns) | 110.9±6.4 (ns) |
| Isobutanol | 51.1±4.2 | 45.1±3.2 (ns) | 60.8±2.2 (*) | 50.4±3.3 (ns) | 51.3±2.7 (ns) |
|  |  |  |  |  |  |
| **B** |  |  |  |  |  |
|  | Empty plasmid | *EAT1*^s52^ | *EAT1*^i9^ | *IMO32*^s52^ | *IMO32*^i9^ |
| Acetaldehyde | 8.7±4.6 | 8.1±3.2 (ns) | 9.0±4.6 (ns) | 10.1±2.1 (ns) | 12.8±1.9 (ns) |
| Ethyl acetate | 59.0±5.1 | 55.0±6.1 (ns) | 142.9±6.0 (***) | 57.9±7.9 (ns) | 58.9±2.4 (ns) |
| Isoamyl acetate | 5.31±0.55 | 5.06±0.72 (ns) | 4.63±1.55 (ns) | 5.75±0.87 (ns) | 5.98±0.33 (ns) |
| Isobutyl acetate | 0.86±0.12 | 0.74±0.09 (ns) | 0.78±0.23 (ns) | 0.84±0.15 (ns) | 0.87±0.03 (ns) |
| Ethyl hexanoate | 0.31±0.03 | 0.31±0.07 (ns) | 0.28±0.09 (ns) | 0.33±0.05 (ns) | 0.34±0.02 (ns) |
| Ethyl octanoate | 0.24±0.03 | 0.23±0.02 (ns) | 0.21±0.05 (ns) | 0.26±0.04 (ns) | 0.27±0.02 (ns) |
| Ethyl decanoate | 0.21±0.04 | 0.19±0.02 (ns) | 0.16±0.07 (ns) | 0.22±0.04 (ns) | 0.25±0.02 (ns) |
| Isoamyl alcohol | 111.2±4.5 | 106.3±7.9 (ns) | 117.6±7.6 (ns) | 116.0±8.9 (ns) | 114.8±12.6 (ns) |
| Isobutanol | 57.4±3.5 | 51.5±5.1 (ns) | 58.2±6.4 (ns) | 56.6±5.4 (ns) | 55.4±5.1 (ns) |
