# Supplementary material for: Polygenic Analysis in Absence of Major Effector ATF1 Unveils Novel Components in Yeast Flavor Ester Biosynthesis
Source: mBio. 2018 Aug 28;9(4):e01279-18. doi: 10.1128/mBio.01279-18 (PMC6113618; doi:10.1128/mBio.01279-18)
Supplement: TABLE S4 [file mbo004184043st4.docx]

SUPPLEMENTARY INFORMATION

**Supplementary Table 4. Aroma compounds produced in fermentations of the engineered industrial Anchor NT112 and Kyokai no. 7 wine and saké yeast strains in industrial-like medium.** Aroma profiles produced in fermentations by **A.** Anchor NT112, **B.** Kyokai no. 7 and **C.** MauriBrew Ale 514 strains engineered with *EAT1* frame-shift and *SNF8* nonsense mutations. The strains were fermented in synthetic Chardonnay grape must (Anchor NT112), YP250-25%(w/v) glucose or malt extract media 15°Brix (MauriBrew Ale 514), all at 18°C with 130 rpm magnetic stirring. The significance of any deviation from the mean of the control strain is indicated as follows: ns p>0.05, *p≤0.05, **p≤0.01, ***p≤0.001. All fermentations were carried out with four replicates and aroma production values are shown +/- s.d.

| **A** |  |  |  |  |
| --- | --- | --- | --- | --- |
|  | NT112 | *eat1*^K179fs^ | *snf8^E148^* | *eat1^K179fs^ snf8^E148^* |
| Acetaldehyde | 4.97±0.90 | 5.36±0.88 (ns) | 7.20±0.77 (ns) | 7.40±0.98 (ns) |
| Ethyl acetate | 57.5±1.0 | 76.2±4.9 (**) | 18.7±1.1 (***) | 21.0±1.7 (***) |
| Isobutyl acetate | 0.02±0.00 | 0.06±0.01 (**) | 0.01±0.01 (ns) | 0.01±0.00 (**) |
| Isoamyl acetate | 1.51±0.19 | 2.69±0.18 (**) | 0.28±0.07 (***) | 0.36±0.07 (***) |
| Ethyl butanoate | 0.28±0.01 | 0.27±0.03 (ns) | 0.25±0.01 (ns) | 0.28±0.02 (ns) |
| Ethyl hexanoate | 0.51±0.07 | 0.53±0.07 (ns) | 0.31±0.06 (*) | 0.38±0.06 (ns) |
| Ethyl octanoate | 0.44±0.07 | 0.42±0.08 (ns) | 0.34±0.05 (ns) | 0.38±0.06 (ns) |
| Ethyl decanoate | 0.40±0.08 | 0.32±0.02 (ns) | 0.25±0.06 (ns) | 0.27±0.05 (ns) |
| Isobutanol | 13.7±1.0 | 20.3±2.3 (*) | 18.2±2.5 (ns) | 21.3±0.7 (***) |
| Isoamyl alcohol | 214.0±11.9 | 195.9±10.4 (ns) | 309.0±67.1 (ns) | 320.2±5.7 (***) |
| IAAc/Alc ratio | 0.007±0.001 | 0.014±0.001 (**) | 0.001±0.000 (***) | 0.001±0.000 (***) |
|  |  |  |  |  |

| **B** |  |  |  |  |
| --- | --- | --- | --- | --- |
|  | Kyokai no. 7 | *eat1*^K179fs^ | *snf8^E148^* | *eat1^K179fs^ snf8^E148^* |
| Acetaldehyde | 14.9±18.4 | 11.5±5.9 (ns) | 10.4±3.6 (ns) | 16.1±5.3 (ns) |
| Ethyl acetate | 60.7±1.6 | 57.4±2.8 (ns) | 46.6±4.2 (**) | 48.2±3.3 (**) |
| Isobutyl acetate | 0.15±0.01 | 0.15±0.01 (ns) | 0.08±0.01 (**) | 0.14±0.01 (ns) |
| Isoamyl acetate | 2.16±0.17 | 2.27±0.17 (ns) | 1.32±0.30 (*) | 1.99±0.23 (ns) |
| Ethyl butanoate | 0.33±0.04 | 0.35±0.02 (ns) | 0.23±0.03 (*) | 0.24±0.02 (ns) |
| Ethyl hexanoate | 0.28±0.02 | 0.31±0.03 (ns) | 0.25±0.02 (ns) | 0.27±0.04 (ns) |
| Ethyl octanoate | 0.24±0.01 | 0.26±0.01 (ns) | 0.19±0.03 (ns) | 0.24±0.04 (ns) |
| Ethyl decanoate | 0.11±0.06 | 0.16±0.05 (ns) | 0.07±0.02 (ns) | 0.18±0.05 (ns) |
| Isobutanol | 35.9±4.9 | 36.4±4.1 (ns) | 26.4±1.5 (ns) | 43.7±6.0 (ns) |
| Isoamyl alcohol | 123.7±27.8 | 117.6±16.8 (ns) | 86.0±10.5 (ns) | 126.5±35.6 (ns) |
| IAAc/Alc ratio | 0.024±0.006 | 0.026±0.005 (ns) | 0.016±0.005 (ns) | 0.017±0.005 (ns) |
|  |  |  |  |  |
| **C** |  |  |  |  |
|  | MauriBrew Ale 514 | *eat1*^K179fs^ | *snf8^E148^* | *eat1^K179fs^ snf8^E148^* |
| Acetaldehyde | 10.2±4.1 | 10.6±2.8 (ns) | 3.4±1.0 (ns) | 5.3±0.7 (ns) |
| Ethyl acetate | 27.5±0.9 | 26.4±2.6 (ns) | 32.9±2.0 (*) | 22.5±0.6 (**) |
| Isobutyl acetate | 0.11±0.01 | 0.15±0.01 (**) | 0.21±0.02 (***) | 0.15±0.01 (**) |
| Isoamyl acetate | 3.49±0.35 | 3.59±0.24 (ns) | 6.73±0.40 (***) | 3.75±0.06 (ns) |
| Ethyl butanoate | 0.18±0.01 | 0.16±0.02 (ns) | 0.18±0.01 (ns) | 0.20±0.01 (ns) |
| Ethyl hexanoate | 0.43±0.03 | 0.35±0.04 (ns) | 0.49±0.03 (ns) | 0.47±0.01 (ns) |
| Ethyl octanoate | 0.51±0.04 | 0.48±0.04 (ns) | 0.72±0.04 (**) | 0.71±0.02 (**) |
| Ethyl decanoate | 0.33±0.03 | 0.34±0.01 (ns) | 0.69±0.03 (***) | 0.68±0.05 (***) |
| Isobutanol | 24.7±0.6 | 32.9±1.2 (***) | 23.9±0.5 (ns) | 27.1±1.2 (ns) |
| Isoamyl alcohol | 137.2±3.8 | 145.0±5.1 (ns) | 135.2±4.4 (ns) | 122.9±3.4 (**) |
| IAAc/Alc ratio | 0.026±0.003 | 0.025±0.002 (ns) | 0.050±0.004 (***) | 0.031±0.001 (ns) |
