# Supplementary material for: Polygenic Analysis in Absence of Major Effector ATF1 Unveils Novel Components in Yeast Flavor Ester Biosynthesis
Source: mBio. 2018 Aug 28;9(4):e01279-18. doi: 10.1128/mBio.01279-18 (PMC6113618; doi:10.1128/mBio.01279-18)
Supplement: TABLE S5 [file mbo004184043st5.docx]

SUPPLEMENTARY INFORMATION

**Supplementary Table 5. Aroma compounds produced in fermentations of the engineered industrial Anchor NT112 wine and Kyokai no. 7 saké yeast strains in industrial-like medium.** Aroma profiles produced in fermentations by **A.** Anchor NT112, **B.** Kyokai no. 7 and **C.** MauriBrew Ale 514 strains engineered with *EAT1* frame-shift and *SNF8* nonsense mutations. The strains were all fermented with YP250-10%(w/v) glucose 25°C with 130 rpm magnetic stirring. The significance of any deviation from the mean of the control strain is indicated as follows: ns p>0.05, *p≤0.05, **p≤0.01, ***p≤0.001. All fermentations were carried out with four replicates and aroma production values are shown +/- s.d.

| **A** |  |  |  |  |
| --- | --- | --- | --- | --- |
|  | NT112 | *eat1*^K179^*^fs^* | *snf8*^E148*^ | *eat1*^K179^*^fs^* *snf8*^E148*^ |
| Acetaldehyde | 2.94±0.32 | 3.48±0.24(ns) | 5.44±0.44(***) | 10.82±0.53(***) |
| Ethyl acetate | 39.17±1.53 | 42.28±1.77(ns) | 33.82±1.45(*) | 22.82±0.80(***) |
| Isobutyl acetate | 0.18±0.11 | 0.30±0.03(ns) | 0.12±0.01(ns) | 0.16±0.02(ns) |
| Isoamyl acetate | 3.21±0.25 | 3.05±0.24(ns) | 2.78±0.19(ns) | 3.63±0.23(ns) |
| Ethyl hexanoate | 0.45±0.04 | 0.30±0.20(ns) | 0.30±0.20(ns) | 0.38±0.26(ns) |
| Ethyl octanoate | 0.82±0.02 | 0.85±0.04(ns) | 0.70±0.04(*) | 0.78±0.03(ns) |
| Ethyl decanoate | 0.94±0.02 | 1.53±0.19(**) | 1.15±0.06(**) | 1.35±0.04(***) |
| Isobutanol | 32.90±2.34 | 36.79±4.57(ns) | 14.73±0.43(***) | 23.71±1.36(**) |
| Isoamyl alcohol | 93.42±5.92 | 82.94±5.43(ns) | 69.21±1.94(**) | 104.10±3.03(ns) |
| IAAT ratio | 0.034±0.003 | 0.037±0.002(ns) | 0.040±0.003(ns) | 0.035±0.001(ns) |

| **B** |  |  |  |  |
| --- | --- | --- | --- | --- |
|  | Kyokai no. 7 | *eat1*^K179^*^fs^* | *snf8*^E148*^ | *eat1*^K179^*^fs^* *snf8*^E148*^ |
| Acetaldehyde | 4.19±0.51 | 2.72±0.64(ns) | 6.48±1.06(ns) | 8.34±2.14(ns) |
| Ethyl acetate | 30.28±0.85 | 33.98±1.19(*) | 28.73±1.13(ns) | 31.86±0.83(ns) |
| Isobutyl acetate | 0.36±0.02 | 0.37±0.02(ns) | 0.29±0.03(*) | 0.37±0.02(ns) |
| Isoamyl acetate | 3.90±0.30 | 4.13±0.42(ns) | 4.52±0.62(ns) | 5.02±0.59(ns) |
| Ethyl hexanoate | 0.32±0.04 | 0.23±0.15(ns) | 0.34±0.23(ns) | 0.24±0.16(ns) |
| Ethyl octanoate | 0.94±0.05 | 0.97±0.02(ns) | 1.00±0.07(ns) | 0.97±0.04(ns) |
| Ethyl decanoate | 1.16±0.16 | 1.36±0.11(ns) | 1.46±0.18(ns) | 1.83±0.14(**) |
| Isobutanol | 48.29±1.46 | 50.70±2.79(ns) | 41.69±1.23(**) | 51.23±5.32(ns) |
| Isoamyl alcohol | 98.26±3.08 | 104.91±5.55(ns) | 114.29±1.31(***) | 124.95±11.05(*) |
| IAAT ratio | 0.040±0.004 | 0.040±0.006(ns) | 0.040±0.005(ns) | 0.041±0.008(ns) |

| **C** |  |  |  |  |
| --- | --- | --- | --- | --- |
|  | MauriBrew Ale 514 | *eat1*^K179^*^fs^* | *snf8*^E148*^ | *eat1*^K179^*^fs^* *snf8*^E148*^ |
| Acetaldehyde | 3.35±1.58 | 2.86±0.31(ns) | 20.16±2.07(***) | 7.57±0.21(*) |
| Ethyl acetate | 25.01±1.90 | 26.29±1.59(ns) | 24.88±1.60(ns) | 25.20±0.94(ns) |
| Isobutyl acetate | 0.11±0.01 | 0.17±0.01(**) | 0.17±0.01(**) | 0.17±0.02(**) |
| Isoamyl acetate | 1.32±0.12 | 1.75±0.24(ns) | 1.53±0.14(ns) | 1.77±0.23(ns) |
| Ethyl hexanoate | 0.28±0.19 | 0.34±0.03(ns) | 0.12±0.24(ns) | 0.45±0.30(ns) |
| Ethyl octanoate | 0.94±0.04 | 0.91±0.05(ns) | 0.64±0.04(***) | 0.77±0.06(*) |
| Ethyl decanoate | 0.83±0.13 | 0.78±0.51(ns) | 0.81±0.05(ns) | 0.95±0.02(ns) |
| Isobutanol | 27.96±1.63 | 40.15±3.21(**) | 26.93±1.16(ns) | 31.41±1.45(ns) |
| Isoamyl alcohol | 74.03±2.62 | 97.55±8.65(*) | 62.94±1.72(**) | 73.76±2.98(ns) |
| IAAT ratio | 0.018±0.002 | 0.018±0.001(ns) | 0.024±0.002(*) | 0.024±0.004(ns) |
