# Supplementary material for: Polygenic Analysis in Absence of Major Effector ATF1 Unveils Novel Components in Yeast Flavor Ester Biosynthesis
Source: mBio. 2018 Aug 28;9(4):e01279-18. doi: 10.1128/mBio.01279-18 (PMC6113618; doi:10.1128/mBio.01279-18)
Supplement: TABLE S6 [file mbo004184043st6.docx]

SUPPLEMENTARY INFORMATION

**Supplementary table 6. Strains used in this study.**

| **Yeast strain** | **Description** | **Source and/or reference** |
| --- | --- | --- |
| TMB 3000 | Low ethyl acetate producing strain, isolated from a paper sludge fermentation plant in Sweden. Diploid | Linden T, Peetre J, Hahn-Hagerdal B. 1992. Isolation and characterization of acetic acid-tolerant galactose-fermenting strains of *Saccharomyces cerevisiae* from a spent sulfite liquor fermentation plant. Appl Environ Microbiol 58:1661-9. |
| Anchor NT112 | High ethyl acetate producing strain, commercial wine yeast. Diploid | Anchor wine yeasts, France |
| TMB3000 *ho*::attL | TMB3000 with a double knockout of the *HO* gene using KanMX and BleMX resistance markers. Resistance markers looped out with phiC31 | This study |
| Anchor NT112 *ho*::attL | Anchor NT112 with a double knockout of the *HO* gene using KanMX and NatMX resistance markers. Resistance markers looped out with phiC31 | This study |
| s25 *ho::attL* | Segregant of TMB 3000 *ho*::attL, MATα | This study |
| s33 *ho::attL* | Segregant of TMB 3000 *ho*::attL, MAT**a** | This study |
| s41 *ho::attL* | Segregant of TMB 3000 *ho*::attL, MAT**a** | This study |
| s52 *ho::attL* | Segregant of TMB 3000 *ho*::attL, MAT**a**. | This study |
| i2 *ho::attL* | Segregant of Anchor NT112 *ho*::attL, MATα | This study |
| i7 *ho::attL* | Segregant of Anchor NT112 *ho*::attL, MAT**a** | This study |
| i8 *ho::attL* | Segregant of Anchor NT112 *ho*::attL, MAT**a** | This study |
| i9 *ho::attL* | Segregant of Anchor NT112 *ho*::attL, MATα. | This study |
| i11 *ho::attL* | Segregant of Anchor NT112 *ho*::attL, MATα | This study |
| s25 *ho::attL atf1*::BleMX | s25 with the *ATF1* gene replaced with a BleMX resistance marker | This study |
| s33 *ho::attL atf1*::BleMX | s25 with the *ATF1* gene replaced with a BleMX resistance marker | This study |
| s41 *ho::attL atf1*::BleMX | s25 with the *ATF1* gene replaced with a BleMX resistance marker | This study |
| s52 *ho::attL atf1*::BleMX | s52 with the *ATF1* gene replaced with a BleMX resistance marker. Haploid parental strain used for QTL mapping | This study |
| i2 *ho::attL atf1*::kanMX | s25 with the *ATF1* gene replaced with a KanMX resistance marker | This study |
| i7 *ho::attL atf1*::kanMX | s25 with the *ATF1* gene replaced with a KanMX resistance marker | This study |
| i8 *ho::attL atf1*::kanMX | s25 with the *ATF1* gene replaced with a KanMX resistance marker | This study |
| i9 *ho::attL atf1*::kanMX | s25 with the *ATF1* gene replaced with a KanMX resistance marker. Haploid parental strain used for QTL mapping | This study |
| i11 *ho::attL atf1*::kanMX | s25 with the *ATF1* gene replaced with a KanMX resistance marker | This study |
| s52 *ho::attL atf1*::BleMX *atf2*::NatMX | s52 with the *ATF1* and *ATF2* genes replaced with BleMX and NatMX resistance markers | This study |
| i9 *ho::attL atf1*::KanMX *atf2*::NatMX | i9 with the *ATF1* and *ATF2* genes replaced with KanMX and NatMX resistance markers | This study |
| s52/i9 *ho*::attL *atf1*:: BleMX/KanMX | Diploid hybrid obtained by crossing s52 *ho*::attL *atf1*::BleMX and i9 *ho*::attL *atf1*::KanMX. Strain used to obtain segregants for QTL mapping with *atf1*Δ pools | This study |
| s52 *ho*::BleMX | s52 with the *HO* gene replaced with a BleMX resistance marker. Used for QTL mapping | This study |
| i9 *ho*::KanMX | i9 with the *HO* gene replaced with a KanMX resistance marker. Haploid parental strain used for QTL mapping | This study |
| s52/i9 *ho*::BleMX/KanMX | Diploid hybrid obtained by crossing s52 *ho*::BleMX and i9 *ho*::KanMX. Strain used to obtain segregants for QTL mapping with WT *ATF1* pools | This study |
| s52/i9 *ho*::attL *atf1*::attL | Diploid hybrid with looped out resistance markers obtained by inducing phiC31 integrase in s52/i9 *ho*::attL *atf1*:: BleMX/KanMX. Strain used for RHA and overexpression of *EAT1* and *IMO32* | This study |
| s52/i9 *ho*::attL | Diploid hybrid with looped out resistance markers obtained by inducing phiC31 integrase in s52/i9 *ho*::attL *atf1*:: BleMX/KanMX. Strain used for RHA and overexpression of *EAT1* and *IMO32* | This study |
| s52/i9 *atf1*Δ p426 | s52/i9 *ho*::attL *atf1*::attL transformed with an empty p426 overexpression plasmid containing a hphMX resistance cassette | This study |
| s52/i9 *atf1*Δ p426_*EAT1*^s52^ | s52/i9 *ho*::attL *atf1*::attL transformed with the p426 overexpression plasmid containing a hphMX resistance cassette, cloned with the *EAT1* gene from s52 expressed under TEF1 promoter and CYC1 terminator | This study |
| s52/i9 *atf1*Δ p426_*EAT1*^i9^ | s52/i9 *ho*::attL *atf1*::attL transformed with the p426 overexpression plasmid containing a hphMX resistance cassette, cloned with the *EAT1* gene from i9 expressed under TEF1 promoter and CYC1 terminator | This study |
| s52/i9 *atf1*Δ p426_*IMO32*^s52^ | s52/i9 *ho*::attL *atf1*::attL transformed with the p426 overexpression plasmid containing a hphMX resistance cassette, cloned with the *IMO32* gene from s52 expressed under TEF1 promoter and CYC1 terminator | This study |
| s52/i9 *atf1*Δ p426_*IMO32*^i9^ | s52/i9 *ho*::attL *atf1*::attL transformed with the p426 overexpression plasmid containing a hphMX resistance cassette, cloned with the *IMO32* gene from i9 expressed under TEF1 promoter and CYC1 terminator | This study |
| s52/i9 p426 | s52/i9 *ho*::attL transformed with an empty p426 overexpression plasmid containing a hphMX resistance cassette | This study |
| s52/i9 p426_*EAT1*^s52^ | s52/i9 *ho*::attL transformed with the p426 overexpression plasmid containing a hphMX resistance cassette, cloned with the *EAT1* gene from s52 expressed under TEF1 promoter and CYC1 terminator | This study |
| s52/i9 p426_*EAT1*^i9^ | s52/i9 *ho*::attL transformed with the p426 overexpression plasmid containing a hphMX resistance cassette, cloned with the *EAT1* gene from i9 expressed under TEF1 promoter and CYC1 terminator | This study |
| s52/i9 p426_*IMO32*^s52^ | s52/i9 *ho*::attL transformed with the p426 overexpression plasmid containing a hphMX resistance cassette, cloned with the *IMO32* gene from s52 expressed under TEF1 promoter and CYC1 terminator | This study |
| s52/i9 p426_*IMO32*^i9^ | s52/i9 *ho*::attL transformed with the p426 overexpression plasmid containing a hphMX resistance cassette, cloned with the *IMO32* gene from i9 expressed under TEF1 promoter and CYC1 terminator | This study |
|  |  | This study |
| s52 *ho*::attL *atf1*::attL | s52 superior haploid. The strain was obtained by inducing phiC31 integrase in s52 *ho::attL atf1*::BleMX to loop out the BleMX resistance marker | This study |
| s52 *atf1*Δ *EAT1^fs^*^197K^ | s52 *ho*::attL *atf1*::attL engineered with reversal of the 531_insA_533 INDEL in *EAT1* (frame-shift mutation, *EAT1^fs^*^197K^) and the synonymous A510G mutation to facilitate CRISPR/Cas9 targeting | This study |
| s52 *atf1*Δ *SNF8*^*148E^ | s52 *ho*::attL *atf1*::attL engineered with reversal of the G442T non-sense mutation in *SNF8* (*SNF8^*148^*^E^) | This study |
| s52 *atf1*Δ  *EAT1^fs^*^197K^ *SNF8*^*148E^ | s52 *ho*::attL *atf1*::attL engineered with reversal of the 531_insA_533 INDEL in *EAT1* (frame-shift mutation, *EAT1^fs^*^197K^) and the synonymous A510G mutation to facilitate CRISPR/Cas9 targeting, as well as the reversal of the G442T non-sense mutation in *SNF8* (*SNF8*^*148E^) | This study |
| s52 *EAT1^fs^*^197K^ | s52 *ho*::attL engineered with reversal of the 531_insA_533 INDEL in *EAT1* (frame-shift mutation, *EAT1^fs^*^197K^) and the synonymous A510G mutation to facilitate CRISPR/Cas9 targeting | This study |
| s52 *SNF8*^*148E^ | s52 *ho*::attL engineered with reversal of the G442T non-sense mutation in *SNF8* (*SNF8^*148^*^E^) | This study |
| s52 *EAT1^fs^*^197K^ *SNF8*^*148E^ | s52 *ho*::attL engineered with reversal of the 531_insA_533 INDEL in *EAT1* (frame-shift mutation, *EAT1^fs^*^197K^) and the synonymous A510G mutation to facilitate CRISPR/Cas9 targeting, as well as the reversal of the G442T non-sense mutation in *SNF8* (*SNF8*^*148E^) | This study |
| i9 *ho*::attL *atf1*::attL | i9 inferior haploid used for CRISPR/Cas9 evaluation. The strain was obtained by inducing phiC31 integrase in i9 *ho::attL atf1*::KanMX to loop out the KanMX resistance marker | This study |
| i9 *atf1*Δ *eat1*^K197^*^fs^* | i9 *ho*::attL *atf1*::attL engineered with the 532delA INDEL in *EAT1* (frame-shift mutation, *eat1*^K197^*^fs^*) and the synonymous A510G mutation to facilitate CRISPR/Cas9 targeting | This study |
| i9 *atf1*Δ *snf8*^K148*^ | i9 *ho*::attL *atf1*::attL engineered with the G442T non-sense mutation in *SNF8* (*snf8*^E148*^) | This study |
| i9 *atf1*Δ  *eat1*^K197^*^fs^* *snf8*^E148*^ | i9 *ho*::attL *atf1*::attL engineered with the 532delA INDEL in *EAT1* (frame-shift mutation, *eat1*^K197^*^fs^*) and the synonymous A510G mutation to facilitate CRISPR/Cas9 targeting, as well as the G442T non-sense mutation in *snf8* (*snf8*^E148*^) | This study |
| i9 *eat1*^K197^*^fs^* | i9 *ho*::attL engineered with the 532delA INDEL in *EAT1* (frame-shift mutation, *eat1*^K197^*^fs^*) and the synonymous A510G mutation to facilitate CRISPR/Cas9 targeting | This study |
| i9 *snf8*^K148*^ | i9 *ho*::attL engineered with the G442T non-sense mutation in *SNF8* (*snf8*^E148*^) | This study |
| i9 *eat1*^K197^*^fs^* *snf8*^E148*^ | i9 *ho*::attL engineered with the 532delA INDEL in *EAT1* (frame-shift mutation, *eat1*^K197^*^fs^*) and the synonymous A510G mutation to facilitate CRISPR/Cas9 targeting, as well as the G442T non-sense mutation in *snf8* (*snf8*^E148*^) | This study |
| Anchor NT112 *eat1*^K197^*^fs^* | The diploid industrial wine yeast Anchor NT112 engineered with the 532delA INDEL in *EAT1* (frame-shift mutation, *eat1*^K197^*^fs^*) and the synonymous A510G mutation to facilitate CRISPR/Cas9 targeting | This study |
| Anchor NT112 *snf8*^K148*^ | The diploid industrial wine yeast Anchor NT112 engineered with G442T non-sense mutation in *SNF8* (*snf8*^E148*^) | This study |
| Anchor NT112 *eat1*^K197^*^fs^* *snf8*^E148*^ | The diploid industrial wine yeast Anchor NT112 engineered with the 532delA INDEL in *EAT1* (frame-shift mutation, *eat1*^K197^*^fs^*) and the synonymous A510G mutation to facilitate CRISPR/Cas9 targeting, as well as the G442T non-sense mutation in *snf8* (*snf8*^E148*^) | This study |
| Kyokai no. 7 | Industrial saké strain. Diploid | The Brewing Society of Japan |
| Kyokai no. 7 *eat1*^K197^*^fs^* | The diploid industrial saké yeast Kyokai no. 7 engineered with the 532delA INDEL in *EAT1* (frame-shift mutation, *eat1*^K197^*^fs^*) and the synonymous A510G mutation to facilitate CRISPR/Cas9 targeting | This study |
| Kyokai no. 7 *snf8*^K148*^ | The diploid industrial saké yeast Kyokai no. 7 engineered with G442T non-sense mutation in *SNF8* (*snf8*^E148*^) | This study |
| Kyokai no. 7 *eat1*^K197^*^fs^* *snf8*^E148*^ | The diploid industrial saké yeast Kyokai no. 7 engineered with the 532delA INDEL in *EAT1* (frame-shift mutation, *eat1*^K197^*^fs^*) and the synonymous A510G mutation to facilitate CRISPR/Cas9 targeting, as well as the G442T non-sense mutation in *snf8* (*snf8*^E148*^) | This study |
| MauriBrew Ale 514 | Industrial ale brewing strain. Diploid | Mauribrew, Australia |
| MauriBrew Ale 514 *eat1*^K197^*^fs^* | The diploid industrial ale brewing yeast MauriBrew Ale 514 engineered with the 532delA INDEL in *EAT1* (frame-shift mutation, *eat1*^K197^*^fs^*) and the synonymous C519T mutation to facilitate CRISPR/Cas9 targeting | This study |
| MauriBrew Ale 514 *snf8*^K148*^ | The diploid industrial saké yeast Kyokai no. 7 engineered with G442T non-sense mutation in *SNF8* (*snf8*^E148*^) | This study |
| MauriBrew Ale 514 *eat1*^K197^*^fs^* *snf8*^E148*^ | The diploid industrial saké yeast Kyokai no. 7 engineered with the 532delA INDEL in *EAT1* (frame-shift mutation, *eat1*^K197^*^fs^*) and the synonymous C519T mutation to facilitate CRISPR/Cas9 targeting, as well as the G442T non-sense mutation in *snf8* (*snf8*^E148*^) | This study |
